# *SLA-1* Genetic Diversity in Pigs: Extensive Analysis of Copy Number Variation, Heterozygosity, Expression, and Breed Specificity

**DOI:** 10.1038/s41598-020-57712-5

**Published:** 2020-01-20

**Authors:** Minh Thong Le, Hojun Choi, Hyejeong Lee, Van Chanh Quy Le, Byeongyong Ahn, Chak-Sum Ho, Kwonho Hong, Hyuk Song, Jin-Hoi Kim, Chankyu Park

**Affiliations:** 1 Department of Stem Cells and Regenerative Biology, Konkuk University, Seoul, 143-701 Korea; 2 Gift of Life Michigan, Ann Arbor, MI 48108 USA; 3 Present Address: School of Biotechnology, International University, Ho Chi Minh City, Vietnam; 4 Vietnam National University, Ho Chi Minh City, Vietnam

**Keywords:** Agricultural genetics, Immunogenetics

## Abstract

Swine leukocyte antigens play indispensable roles in immune responses by recognizing a large number of foreign antigens and thus, their genetic diversity plays a critical role in their functions. In this study, we developed a new high-resolution typing method for pig *SLA-1* and successfully typed 307 individuals from diverse genetic backgrounds including 11 pure breeds, 1 cross bred, and 12 cell lines. We identified a total of 52 alleles including 18 novel alleles and 9 *SLA-1* duplication haplotypes, including 4 new haplotypes. We observed significant differences in the distribution of *SLA-1* alleles among the different pig breeds, including the breed specific alleles. *SLA-1* duplication was observed in 33% of the chromosomes and was especially high in the biomedical model breeds such as SNU (100%) and NIH (76%) miniature pigs. Our analysis showed that *SLA-1* duplication is associated with the increased level of *SLA-1* mRNA expression in porcine cells compared to that of the single copy haplotype. Therefore, we provide here the results of the most extensive genetic analysis on pig *SLA-1*.

## Introduction

Pigs are invaluable as a species for meat production and as experimental models in biomedical research^1–3^. In addition, the increase in the occurrence of infectious diseases has become a big concern for the pig production industry^4^. The swine major histocompatibility complex (MHC) namely swine leukocyte antigen (SLA), has been associated with the porcine immune response to various infections and vaccinations^5–7^. Several QTLs also have been mapped to the SLA region including antibody response to porcine reproductive and respiratory syndrome (PRRS)^8^.

The major histocompatibility complex (MHC) recognizes antigens and activates the immune reactions^9,10^. MHC polymorphisms play an essential role in determining the functional specificity of the molecules to antigens^7,11^. Therefore, comprehensive identification and characterization of the alleles of major MHC genes are important to predict adaptive immune responses of an individual. In the humans, about 12,000 alleles of MHC genes have been reported^12^. Currently, only 227 and 192 alleles have been reported for SLA class I and II genes in IPD-MHC database (https://www.ebi.ac.uk/ipd/mhc/group/SLA/). Therefore, further efforts are necessary to characterize the allelic diversity of major SLA genes.

Several methods have been used to investigate the genetic diversity of SLA genes, including the polymerase chain reaction sequence-specific primer methods (PCR-SSP)^13–15^, PCR-restriction fragment length polymorphism (PCR-RFLP)^13,16^, and cDNA based typing^17^. However, these DNA based typing methods still require further improvement in the resolution of the typing results, comprehensiveness in allele coverage, and usability for large-scale typing. To improve these, we previously developed the genomic sequence-based high-resolution typing (GSBT) methods for *SLA-2*, -*DQA*, -*DQB1*, and -*DRB1*, and presented the results of new allele identification using a large number of field samples and population genetic analysis on diverse breeds^18–22^

However, the precise typing of *SLA-1* has been particularly difficult because of the presence of a large number of novel alleles and copy number variations (CNVs) of the locus. Thus, the development of a robust typing method for *SLA-1* is necessary. Currently, 89 alleles for *SLA-1* have been reported in the IPD database including the results from this study^23^.

*SLA-1*, *SLA-2*, and *SLA-3* are constitutively expressed classical *MHC class I* genes, but their expression may vary depending on the genetic differences. For example, *SLA-1* is duplicated in the haplotypes Hp-2.0, Hp-8.0, Hp-11.0, Hp-12.0, Hp-19.0, Hp-20.0, and Hp-27.0^14,24–26^. In addition, *SLA-1*, *3*, and 6 were not expressed in the haplotypes Hp-3.0, Hp-2.0, and Hp-5.0, respectively^24^. Recently, a method was reported to estimate the copy number of *SLA-1* and to facilitate our understanding on the functional aspect of *SLA-1* duplication^27^. The frequency of *SLA-1* duplication could be abundant, but the detailed functional analysis is not been available.

Therefore, we developed a genomic DNA based high resolution *SLA-1* typing method with high accuracy regardless of CNVs and present the extensive analysis results of *SLA-1* diversity including new alleles and haplotypes, and allelic distribution among different breeds. We also analyzed the level of *SLA-1* expression in pig cells according to their copy numbers which could affect MHC class I-specific immune responses. The information presented in this study should contribute to improving our understanding on the genetic polymorphisms of *SLA-1* in diverse pig breeds.

## Results

### Determination of the SLA-1 specific region

**Figure 1:**
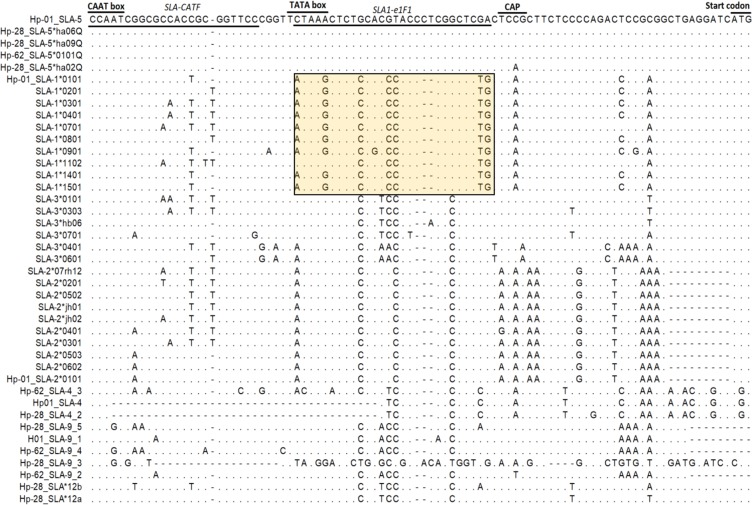
Comparison of the nucleotide sequence variations in the 5′-UTR region among the seven swine leukocyte antigen (*SLA*) classical class I-related genes. An alignment using 41 sequences of the 92-bp region containing CAT box, TATA box, CAP region from *SLA-1, -2, -3, -4, -5, -9* and -*12* is shown. The primer positions of SLA-CATF, the forward primers used for the co-amplification of *SLA classical class I* gene, and SLA1-e1F1, SLA-1-specific amplification, are underlined. *SLA-1* specific regions are indicated in a rectangle. The identical and missing nucleotides are indicated by dots (.) and dashes (–), respectively.

To develop a genomic DNA-based typing method of *SLA-1*, the determination of conserved locus specific region to design *SLA-1* specific primers is required. We previously reported the locus specific nucleotide sequence variations at the downstream promoter region from six classical *SLA* class I-related genes including *SLA-1, -2, -3, -4, -5*, and *-9*^21^. Here, we extended the results by incorporating genomic sequences from additional cloning and sequence analysis. As a result, we identified a *SLA-1* specific motif between the TATA box and the CAP site in the 5ʹ UTR and designed a *SLA-1*-specific forward direction primer, SLA1-e1F1 (Table 1), from an alignment with 41 unique sequences of *SLA* classical class I-like genes consisting of 10 *SLA-1*, 10 *SLA-2*, 6 *SLA-3*, 3 *SLA-4*, 5 *SLA-5*, 5 *SLA-9*, and 2 *SLA-*12 (Fig. 1). By combining SLA1-e1F1 together with SLA-e4R4, which is the previously developed classical *SLA* class I gene specific reverse primer^21^, we successfully obtained 1844-bp *SLA-1* amplicons from 9 selected samples consisting of different breeds and cell lines showing high genetic diversity (Fig. S1).

**Table 1:** Primer sequences used in this study.

	Primer sequence (5′-3′)	Target region	Use	Annealing temperature (oC)	Product size (bp)
SLA1-e1F1	MTAARCTCTCCRCCCASCCGGCTCTG	5′ UTR	Typing PCR	65	1844
SLA-e4R4	cGGGTCACATGTGTCyTTGGAGG	Exon 4			
SLA1-e1F1	MTAARCTCTCCRCCCASCCGGCTCTG	5′ UTR	Real time PCR	65	137
SLA-e12R	AGGGAGTGGGGACCCGCCT	Exon 1–2			
GAPDH-F2	CCTGGCCAAGGTCATCCA	Exon 6	Real time PCR	53	123
GAPDH-R2	CGGCCATCACGCCACAG	Exon 7			
SLA-CATF	CCAATC**R**GCG**MM**AC**Y**GCTGGTTCC	5′ UTR	Cloning for primer design	65	1939
SLA-R	GATCTCCTTAGGGTAGAAGCCCAaatta CAGCACCTCA	Exon 4			
LSPE2F	CSTGTCCCGGCCCGAC	Exon 2	Cloning sequencing	50	
SLA-CQ2R	TTCCTGGGGATGGGGATG	Intron 2			
LSPE3F	GCGGGGTCAGGGTCTC	Exon 3			
SLA-i3R2	GAGGGGAGATGGTGGAG	Intron 3			
SLA1-Seq. 2-F	tgctatgctgtgCGCCGA**R**AGGAGGGT	Intron 1	Typing sequencing		
SLA1-Seq. 2-R	ACCCGGAGGTCGGGGT	Intron 2			
SLA1-Seq. 3-F	atgctgattatcgCCC**K**GGTTGG**W**CGCG	Intron 2			
SLA1-Seq. 3-R	TCCTCCCTCTCAGGACAG	Intron 3			
T7	TAATACGACTCACTATAGGG	cloning vector	Cloning sequencing		
T3	ATTAACCCTCACTAAAG				
SP6	ATTTAGGTGACACTATAG				

Note: Nucleotides in bold indicate degenerate bases. Sequences in lower case indicate the non-template-based regions to improve PCR and sequencing efficiency.

### Development of a genomic DNA-based high-resolution SLA-1 typing

**Figure 2:**
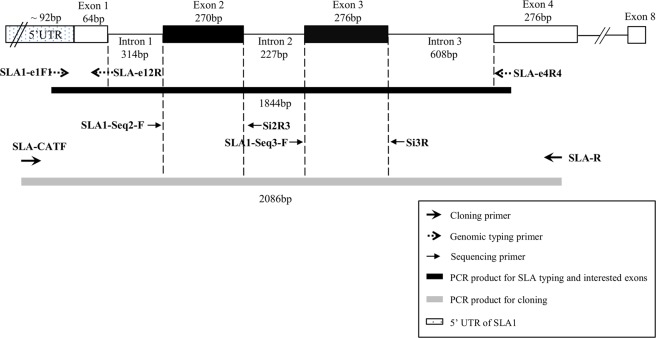
General overview of the genomic sequence-based *SLA-1* typing method. The diagram shows the location and directions of primers used for polymerase chain reaction (PCR) amplification and sequencing. The sizes (bp) of introns, exons, and PCR products are indicated. Because the upstream boundary of the 5′ untranslated region of *SLA-1* was unknown, only the minimum size (>92 bp) is indicated.

The complete coverage of *SLA-1* exons 2 and 3 sequence information is the minimum requirement to officially assign alleles of *SLA class I* genes^28^. It has been proven that combining the locus-specific PCR and subsequent direct sequencing using independent primers is a successful method for the comprehensive typing of hyper polymorphic *MHC* genes^18,20–22^. Taking the advantage of the small sizes of the introns surrounding the exons 2 and 3, we succeeded in comprehensively amplifying the 1844-bp *SLA-1*-specific amplicons from all the tested samples (Fig. 2). However, the direct sequencing of heterozygous amplicons carrying the nucleotide deletion and non-deletion alleles (namely deletion heterozygotes) at the intronic regions often resulted in unresolvable chromatograms. To address the problem, we characterized indels at introns 1, 2, and 3 of diverse *SLA-1* alleles including SLA-1*01:01, 02:01, 03:01, 04:01, 07:01, 08:01, 09:01, 14:01, and 15:01 by cloning and sequence analysis (Fig. S2). From the analysis, we identified three deletions located at −41 and −42 bp upstream (intron 1) of exon 2, +13 bp downstream (intron 2) of exon 2, and +24 bp downstream (intron 3) of exon 3, respectively.

Through reiterative primer design and sequencing, we finally developed the sequencing primers, SLA1-seq2-F and SLA1-seq3-F, which generated clear sequencing results of *SLA-1* exons 2 and 3 even from the deletion heterozygotes (Table 1, Fig. 2, Fig. S2). For sequencing from the reverse direction to confirm new alleles, we also developed the primers Si2R3 and Si3R, suitable for the direct sequencing of the homozygotes. However, cloning was required for the deletion heterozygotes for successful sequencing (Fig. S2). Consequently, we obtained high resolution typing results of *SLA-1* from the genomic DNA of 307 individuals from 14 sample sets without any failure (Table S1), demonstrating the successful development of genomic DNA-based comprehensive high-resolution *SLA-1* typing.

### Confirmation of the accuracy of new SLA-1 genomic sequence-based typing (GSBT)

To validate the accuracy of our typing results using *SLA-1* GSBT, we firstly investigated the presence of conflicts in Mendelian segregation from the typing results of nine KNP families (39 pigs) and two KNP x Landrace cross families (10 pigs) (Table S2). Nine *SLA-1* alleles from 13 homozygotes and 36 heterozygotes were observed with complete agreement with Mendelian segregation. Secondly, typing eight ATCC pig cell lines identified 15 alleles which cover 9 subgroups and the results were consistent with those previously reported^14,29^. In addition, the primers SLA1-e1F1 and SLA-e4R4 also generated *SLA-1* specific 727-bp amplicons from the cDNA, in addition to genomic PCR because they are located on exonal regions. Therefore, reverse transcription PCR using the primers and the subsequent direct sequencing results in the successful typing for *SLA-1*, showing a complete agreement between the *SLA-1* GSBT and cDNA typing (Table S3). Lastly, we carried out blind sample testing (n = 40) in collaboration with the ISAG nomenclature committee. The results concorded with the expected results, excepting the new alleles additionally identified from our typing results (data not shown), supporting the accuracy and comprehensiveness of our new *SLA-1* typing method.

### Identification of novel alleles of SLA-1

A total of 52 *SLA-1* alleles corresponding to 34 IPD-SLA curated, 8 NCBI noncurated, and 10 novel sequences were detected from the typing of 307 samples (Tables 1–3). The new alleles were confirmed by cloning and bidirectional sequencing. Among the new alleles, SLA-1*19:02 and *23:03 were observed only once in the Lanyu and Ossabaw pigs probably because of the sample size limit. The remainder were observed from at least two individuals (Table S4). The allele names for new and non-curated NCBI alleles were assigned by the ISAG-SLA nomenclature committee and submitted to IPD (Table S4). The new alleles were clustered into 7 existing subgroups (SLA-1*07, 08, 15, 16, 18, 19, and 20) and formed two new subgroups (SLA-1* 21 and 23) (Fig. S3).

**Table 2:** The distribution of allele frequencies of *SLA-1* among different pig breeds.

Allele	Accession	KNP (114)^d^	SNU (52)	NIH (29)	Duroc (21)	Berkshire (19)	Yorkshire (19)	Landrace (19)	ATCC (8)	Land x KNP (6)	Lanyu (5)	Ossabaw (5)	Meishan (4)	Local PAM cells (4)	AGH (2)	All (307)
01:01	AK236893	0^e^	0	0	0	0	10.2	4.65	0	0	0	0	0	0	0	0.86
02:01	AY135592	0	43.27	43.14	0	0	0	0	0	0	0	0	0	0	0	16.38
02:02	EU440334	0	0	0	0	0	8.16	0	10.53	0	0	0	15.38	0	0	0.98
04:01	AK396599	0	0	13.73	73.81	1.89	4.08	4.65	21.05	0	90	0	30.77	0	25	8.31
04:02	AK398003	0	0	0	0	3.77	0	0	0	0	0	0	0	25	0	0.49
06:01	AK398228	0	6.73	0	0	0	0	0	0	0	0	0	0	0	0	1.71
07:01	EU440339	0	43.27	43.14	0	0	0	0	5.26	0	0	0	0	0	0	16.5
07:02	AY135587	0	0	0	2.38	1.89	0	2.33	5.26	0	0	0	0	12.5	0	0.61
07:03^a^	KU754555	0	0	0	0	0	2.04	0	0	0	0	0	0	0	0	0.12
07:04	KU754554	0	0	0	0	0	2.04	0	0	0	0	0	0	0	0	0.12
07:05	EU440331	0	0	0	0	0	0	0	5.26	0	0	0	0	0	0	0.12
07:07^b^	MF871653	0	0	0	0	0	0	0	0	0	0	0	0	0	50	0.24
07:08^b^	MF871650	0	0	0	0	0	8.16	0	0	0	0	0	0	0	0	0.49
08:01	NM001246245	28.22	0	0	0	0	0	4.65	0	14.29	0	0	0	12.5	0	8.92
08:03	AF464043	0	0	0	0	0	0	4.65	0	0	0	0	0	0	0	0.24
08:04	AK396218	0	0	0	0	0	0	0	0	0	0	33.33	0	0	0	0.49
08:05	AF464015	0	0	0	0	0	0	2.33	0	0	0	0	0	0	0	0.12
08:07	KU953375	0	0	0	0	0	0	0	5.26	0	0	0	0	0	0	0.12
08:08	EU440332	0	0	0	0	0	8.16	0	5.26	0	0	0	0	0	0	0.61
08:10	AK231553	0	0	0	0	0	16.33	2.33	0	0	0	0	0	12.5	0	1.22
08:11	AK351685	0	0	0	0	0	2.04	6.98	0	0	0	0	0	0	0	0.49
08:13	AY459299	0	0	0	0	0	0	0	0	0	0	0	7.69	0	0	0.12
08:15^a^	KJ555020	0	0	0	0	11.32	0	0	0	0	0	0	0	0	0	0.73
08:16^b^	MF871654	0	0	0	0	0	0	0	0	0	0	25	0	0	0	0.37
08:17^b^	MF871647	0	0	0	16.67	0	0	2.33	0	0	0	0	0	12.5	0	1.1
09:01	AP009556	0	0	0	0	0	0	4.65	0	7.14	0	0	0	0	0	0.37
10:02	DQ303230	0	0	0	0	0	0	0	0	0	0	0	15.38	0	0	0.24
11:01(01|02)^c^	AK396545	0	0	0	0	5.66	2.04	0	10.53	0	0	0	0	0	0	0.73
11:02	DQ992488	40.66	0	0	4.76	0	0	0	0	21.43	0	0	0	25	0	12.84
11:03	DQ883209	5.39	0	0	0	0	8.16	0	0	7.14	0	0	0	0	0	2.2
11:04	EU440338	0	0	0	0	7.55	0	4.65	5.26	0	0	25	0	0	0	1.22
12:01	KC510996	6.64	0	0	0	0	4.08	2.33	5.26	0	0	0	0	0	0	2.44
13:01	AK237395	0	0	0	0	0	4.08	2.33	5.26	0	0	0	0	0	0	0.49
13:02	AY459297	0	0	0	0	0	0	0	0	0	0	0	7.69	0	0	0.12
14:01	EU440343	0	0	0	0	1.89	0	20.93	5.26	21.43	0	0	0	0	0	1.71
14:02	EU440342	0	0	0	0	0	0	0	5.26	0	0	0	0	0	0	0.12
15:01	AK398067	1.24	0	0	0	0	0	9.3	0	7.14	0	0	0	0	0	0.98
15:02	AK346419	12.45	0	0	0	0	0	6.98	0	14.29	0	0	0	0	0	4.28
15:03^a^	MF498783	0	0	0	0	9.43	0	0	0	0	0	0	7.69	0	0	0.73
16:01	MF871646	0	0	0	2.38	0	0	0	0	0	0	0	0	0	25	0.24
17:01	DQ303229	0	0	0	0	0	0	0	0	0	0	0	15.38	0	0	0.24
18:01	EU440333	0	0	0	0	0	8.16	0	5.26	0	0	0	0	0	0	0.61
18:02^a^	AB845314	0	6.73	0	0	0	0	0	0	0	0	0	0	0	0	1.71
19:02^b^	MF871652	0	0	0	0	0	0	0	0	0	10	0	0	0	0	0.12
20:02^b^	MF871649	0	0	0	0	0	0	2.33	0	0	0	0	0	0	0	0.12
21:01^a^	KU754556	5.39	0	0	0	0	6.12	0	0	7.14	0	0	0	0	0	2.08
21:02^b^	MF871648	0	0	0	0	22.64	0	0	0	0	0	8.33	0	0	0	1.59
21:03^a^	AK394788	0	0	0	0	0	4.08	2.33	0	0	0	0	0	0	0	0.37
23:01^a^	KF026021	0	0	0	0	0	0	9.3	0	0	0	0	0	0	0	0.49
23:02^b^	MF871651	0	0	0	0	22.64	0	0	0	0	0	0	0	0	0	1.47
23:03^b^	MF871655	0	0	0	0	0	0	0	0	0	0	8.33	0	0	0	0.12
23:04^a^	KJ555027	0	0	0	0	11.32	2.04	0	0	0	0	0	0	0	0	0.86

^a^Alleles identified by other labs previously, but not reported to IPD.

^b^Novel alleles identified in this study.

^c^The sequences of exons 2 and 3 are identical between the alleles 11:01:01 and 11:01:02. Six-digit allele naming is used to indicate nucleotide difference at exon 4.

^d^The number of animals for each breed.

^e^Allele frequencies.

**Table 3:** Identified allelic linkages by *SLA-1* duplication.

Haplotypes*	Number of pigs	Breeds	Haplotypes	References
23:02–21:02	10	Berk	New	
12:01–13:01	3	York, Land, ATCC	Hp-35.23	Gao *et al*., 2017
02:02–18:01	5	York, ATCC	New	
11:03–21:01	8	York, KNP	New	
02:01–07:01	32	SNU, NIH, ATCC	HP-2.0	Ho *et al*., 2009
06:01–18:02	8	SNU	New	
10:02–17:01	2	Meishan	Hp-20.18	Ho *et al*., 2006
08:13–13:02	1	Meishan	Hp-19.15	Ho *et al*., 2006
15:01–09:01	3	Land, Land x KNP	Hp-28.8b	Gao *et al*., 2017

*Haplotypes indicate allelic linkages between two duplicated *SLA-1* alleles on the same chromosome. The linkage is indicated with a dash (–).

### Identification of new SLA-1 duplication haplotypes

We identified 123 cases of *SLA-1* duplication-bearing typing results from the typing of 307 samples, and they were classified into 9 groups either belonging to previously reported haplotypes (Hp-2, 19, 20, 28, and 35)^28,30^ or new *SLA-1* haplotypes (SLA-1*21:02–23:02 11:03–21:01, 06:01–18:02 and 02:02–18:01 in which two linked *SLA-1* loci are indicated by a dash ‘–’) detected for the first time in this study (Table 3, Table S1). SLA-1*02:02–18:01 was a new combination of previously reported alleles and the remainder were associated with new alleles (Table 3). We also observed 9 additional cases of *SLA-1* duplication, but were unable to separate them into individual haplotypes because the numbers of cases were not enough to determine their haplotypic phases (Table S1).

### Copy number variation of SLA-1 among different pig breeds

We observed *SLA-1* duplication in at least one chromosome from 40.06% (123 pig out of 307) of our typing results (Table S1). In detail, the frequencies of the duplication heterozygotes (4 alleles or duplication in both chromosomes), duplication hemizygotes (3 alleles or duplication in a single chromosome), and duplication homozygotes (2 alleles from duplication in both chromosomes but homozygotes) were 2.93% (n = 9), 13.68% (n = 42), and 23.45% (n = 72), respectively (Table S1). To estimate the frequency of chromosomes with *SLA-1* duplication, we assigned values 1.0, 1.5, and 2.0 to each typing result for no duplication, duplication in only one chromosome, and duplication in both chromosomes, respectively. Then, the average value was 1.33 (204/614), indicating that 33% of chromosomes contains *SLA-1* duplication (Table 4, Table S1).

**Table 4:** Duplication, heterozygosity and diversity of *SLA-1* for various breeds of pigs.

Breeds	Number of samples	Number of duplicated chromosomes	^a^Duplication rate	^b^Observed heterozygosity	^c^Expected heterozygosity	Shannon Index	Evenness
KNP	114	13	0.06	0.64	0.73	1.57	0.78
SNU	52	104	1	1	0.62	1.57	0.78
NIH	29	44	0.76	0.83	0.61	1.44	0.91
Duroc	21	0	0	0.33	0.42	1.22	0.53
Berkshire	19	15	0.39	0.89	0.85	3.02	0.87
Yorkshire	19	11	0.29	0.89	0.92	3.81	0.93
Landrace	19	5	0.13	0.84	0.91	3.89	0.92
ATCC	8	3	—^d^	—	—	—	—
Land x KNP	6	2	—	—	—	—	—
Lanyu	5	0	—	—	—	—	—
Ossabaw	5	2	—	—	—	—	—
Meishan	4	5	—	—	—	—	—
Local PAM cells	4	0	—	—	—	—	—
AGH	2	0	—	—	—	—	—
All	307	204	0.33	0.75	0.91		

^a^Duplication rate = number of chromosomes containing *SLA-1* duplication/total number of chromosomes.

^b^Observed heterozygosity = number of heterozygotes/total number of individuals.

^c^Expected heterozygosity = 1 − ∑ p_i_^2^, where p_i_ is the allele frequency of the *i-*th allele

^d^Values were not calculated due to low numbers (<10) of individuals in the population.

*SLA-1* duplication was observed from most of the breeds except for Duroc, Lanyu, and AGH in this study. The result could be affected by the sample size limit in those breeds. Indeed, *SLA-1* duplication was reported from Duroc, previously^28^. When the frequency of *SLA-1* duplication was compared among breeds, the highest was in Berkshire (39%), followed by Yorkshire (29%), Landrace (13%), and KNP (6%) (Table 4). We also observed the presence of breed-specific duplication haplotypes in which SLA-1*21:02–23:02, 06:01–16:02 and 10:02–17:01 and 08:13–13:02 were only observed in Berkshire, SNU, and Meishan, respectively. The remainder of the haplotypes were shared among the breeds (Table 3).

### High SLA-1 heterozygosity in inbred pigs for biomedical study due to locus duplication

*SLA-1* duplication prevents simple interpretation of typing results in determining allelic combinations. Therefore, our definition for *SLA-1* heterozygosity was simply based on the presence of more than one allele in the typing results regardless of the locus duplication. The average observed heterozygosity of *SLA-1* from our typing results was 51% (n = 158, out of 307) which is affected by a high frequency (54.07%) of inbred pigs such as SNU and NIH in our samples (Table 4, Table S1). However, the values were much higher in the commercial breeds such as Berkshire (89%), Yorkshire (89%), and Landrace (84%) except for Duroc (33%), than the inbred pigs. Interestingly, the observed heterozygosity of the SNU miniature pigs was 100% (n = 52). Further analysis showed that all the *SLA-1* alleles in the SNU miniature pigs were associated with *SLA-1* duplication (Table S1). The result indicates that *SLA-1* duplication can contribute to maintaining the functional heterozygosity in inbred animals (Table 4, Table S1).

### Comparison of SLA-1 genetic diversity among different pig breeds

When we compared the distribution of *SLA-1* alleles across different breed groups with ≥15 individuals in our typing, the allelic constitution differs significantly among the breeds (Table 4). The allelic diversity was the lowest in the NIH miniature pigs with only three, following by the SNU, Duroc, and KNP pigs with 4, 5, and 7 alleles, respectively. Landrace had the highest with 19 alleles, followed by Yorkshire (n = 17), and Berkshire (n = 11), indicating a high genetic diversity of *SLA-1* within these breeds, although the number of analyzed animals were much smaller than the inbred pigs. Interestingly, we observed 14 *SLA-1* alleles from the 8 ATCC pig cell lines, indicating their diverse origins.

In regard to the allelic dominance or prevalence, SLA-1*07:01 and 02:01 were the most abundant with 16.5% and 16.38%, respectively, followed by 11:02 (12.84%), 08:01 (8.92%), and 04:01 (8.31%). However, their frequencies were influenced by the large sample size of SNU (n = 52) and KNP (n = 114), and their allelic dominance in the breeds. In contrast, 11 alleles including 07:04, 07:05, 08:05, 08:07, 08:13, 13:02, 14:02, 20:02, 07:03, 23:03, and 19:02 were detected only once from the typing results of local breeds with limited sample sizes including Lanyu, Ossabaw, Meishan, and AGH (Table 2, Table S1).

**Figure 3:**
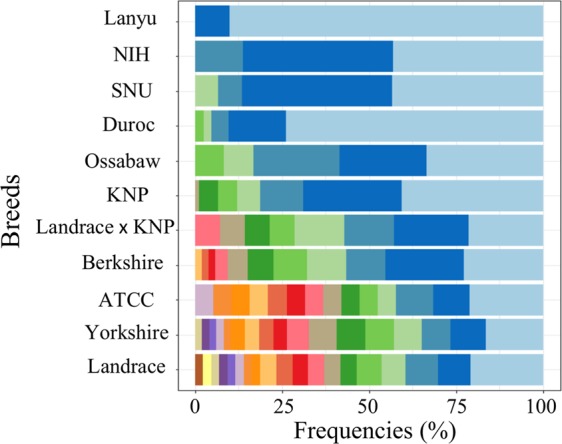
A diagram showing the patterns of allelic diversity and evenness of *SLA-1* among the diverse breeds used in this study. The X-axis shows the frequencies of each allele in the horizontally stacked bar chart, within each breed (Y-axis). The results from the breeds with >5 individuals were used. Note that the color does not indicate the same allele.

The allelic distribution pattern of *SLA-1* across breeds was divided into two groups, breeds showing either dominant or balanced allelic distributions (Fig. 3). Among the 11 breeds with sample sizes ≥5, Duroc, NIH, and Lanyu showed allelic dominance with 04:01 (73.81%), 02:01–07:01 (86.28%), and 04:01 (90%), respectively. In contrast, the rest of the breeds showed a more balanced distribution of alleles in their frequencies than the former. Consistently, the genotypic richness which is the number of genotypes that would be expected was low for NIH (1.44), KNP (1.57), and SNU (1.57) while the values were much higher for Landrace (3.89), Yorkshire (3.81), and Berkshire (3.02) (Table 4). The high genotypic richness may indicate the sign of balancing of selection which results in maintaining the gene pool diversity. Consistently, genotypic evenness in which equally abundant genotypes yields a value equal to 1 was high for Landrace (0.92), Yorkshire (0.93), and Berkshire (0.87). NIH miniature pigs with only three alleles also showed high evenness (0.91) due to the allelic frequency balance among three alleles (Table 4). The allelic diversity of *SLA-1* was low in Duroc with the genetic richness of 1.22 and evenness of 0.53 (Table 4), which is consistent to the results of other *SLA* genes in previous studies including *SLA-2*, *-DQB1*, and *-DRB1*^18,20,21^.

### Low allele sharing in SLA-1 across pig breeds

Pair-wise genetic identity analysis (Nei’s) for *SLA-1* among breeds with sample sizes >10 showed distant relationships among them, except between the SNU and NIH miniature pigs (Table S5) which share the same ancestral origin as the Minnesota miniature pigs^31^. Interestingly, the result of allele sharing analysis using 38 *SLA-1* alleles from six selected breeds (KNP, NIH, Duroc, Berkshire, Yorkshire, Landrace) also showed the presence of no common allele across all six breeds (Fig. S4). SLA-1*04:01 was the most common allele shared across 5 breeds (Berkshire, Duroc, Yorkshire, Landrace, and NIH) (Fig. S4, Table S6). This finding is consistent with previous studies which reported the presence of 04:01 in various other rare breeds including the Micromini pigs, Clawn, Yucatan, and Mexican hairless mini-pigs^24,28,32^. Allele 07:02 was common for Duroc, Berkshire, and Landrace, and 12:01 for KNP, Yorkshire, and Landrace (Table S6). In the remaining alleles, 16 were shared by only 2 breeds (Table S6) and 19 were breed specific (Table S7). Yorkshire harbors the largest number of breed-specific alleles (n = 6). Duroc showed only a single breed-specific allele. Taken together, our results showed the presence of significant genetic difference in *SLA-1* among the different breeds of pigs.

### Increased level of SLA-1 mRNA expression by gene duplication

**Figure 4:**
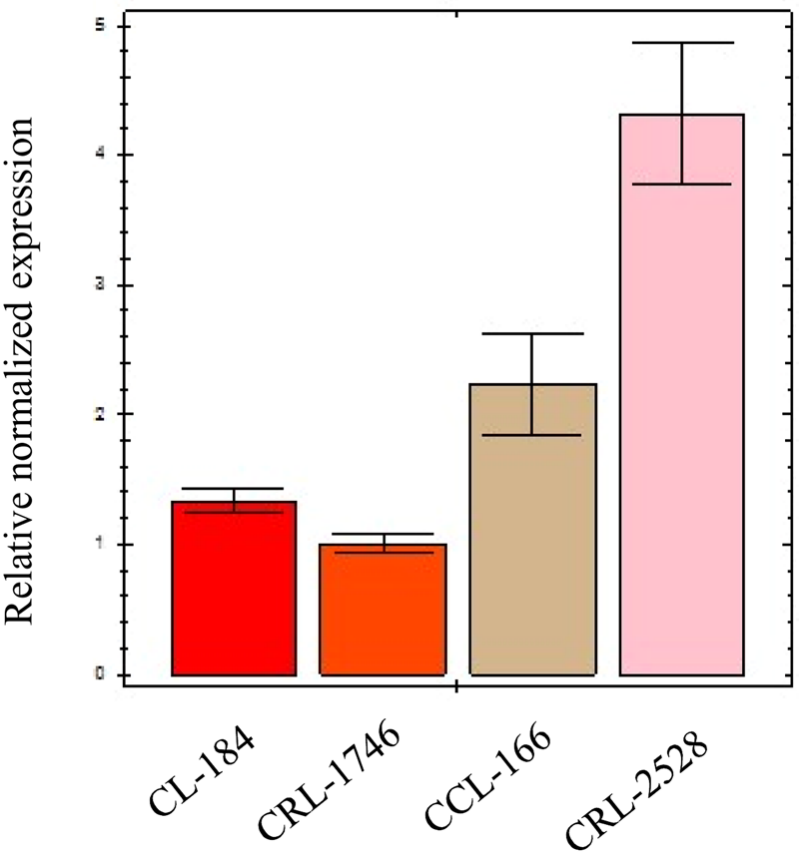
Comparison of the expression levels of *SLA-1* between cells with different *SLA-1* copy numbers. Four fibroblast cell lines of pigs were evaluated for each of different *SLA-1* copy numbers, CL-184 and CRL-1746 for 2 copies, and CCL-166 and CRL-2528 for 3 copies. Cell names are indicated in the bottom.

The relationship between *SLA-1* copy numbers and corresponding expression levels has not been clearly illuminated. Therefore, we identified pig cells with different *SLA-1* copy numbers and evaluated their expression level using real-time PCR (Fig. 4). The average level of *SLA-1* transcripts from cells with three copies of *SLA-1* (CCL-166, CRL-2528) was 2.8 times higher than those with two copies (CL-184 and CRL-1746, p = 0.0025), suggesting a possible importance of *SLA-1* duplication for immune responses in pigs. We also observed variations in the level of *SLA-1* expression in cells with identical copy numbers. This could be resulted from the difference in the characteristics of cells or alleles (Table S3). However, we were unable to conclude the difference in the expression level of *SLA-1* on the protein level among the cells with different *SLA-1* copy numbers because the signals were not only from *SLA-1* but also from *SLA-2* and *-3* due to the unavailability of *SLA-1*-specific antibodies.

## Discussion

The complexity of the *SLA* class I region caused by extreme intra-locus sequence variations and inter-locus sequence similarity, presence of pseudogenes, and locus duplication makes the region difficult to study. To overcome the difficulty, we systemically developed a genomic DNA-based high resolution typing method for *SLA-1* and presented the analysis results from a large-scale typing on diverse pig breeds including the population level analysis of *SLA-1* CNVs.

Considering the existence of over 700 pig breeds or lines worldwide^2^, the number of uncovered alleles still could be significant in numbers. Considering the extreme polymorphisms of *SLA-1*, unraveling the information will not only reveal the genetic diversity of the gene but also contribute to the illumination of the immunogenetic aspects of pig evolution and relationships among them during breed formation^33^.

SLA-1*04:01 was shared by multiple breeds and suggests the early origin of this allele in pigs and the possible importance of the allele in antigen recognition. However, a large number of breed specific alleles (19 alleles, 36.54%) observed in this study may also indicate the influence of recent artificial selections through animal breeding or barriers in gene transfer among the breeds.

*SLA-2* also showed a lower allele sharing among breeds with a large number (n = 28) of breed-specific alleles^21^. The allele SLA-2*04:01 was the most shared allele for *SLA-2* among the major pig breeds and belongs to the same haplotype as SLA-1*04:01 (Hp-4)^28^. This indicated the maintenance of this major class I haplotype during breed diversification. The pattern, however, differs in *SLA class II* genes such as *SLA-DRB1* and *DQB1*. The much lower rate of breed-specific alleles was identified from both *SLA-DRB1* and *DQB1*, than in *SLA class I* genes^18,20^. This could be associated with less polymorphic nature of *SLA* class II genes in comparison with class I genes, suggesting that the analysis of *SLA* class I genes may harbor more information to present the history of the species than *SLA* class II genes.

The MHC class I region appears to have undergone repeated duplication and loss^34^, resulting in three functional classical class I genes in most mammals^35^. However, *SLA-1* was further duplicated in certain haplotypes and more than three functional *MHC class I* genes are present in individuals with the duplicated *SLA-1*. Duplication of *SLA-1* was previously reported from Duroc, Sinclair, Hanford, Westran, Belgian, Danish, Yucatan, Kenyan, and Bama pigs, indicating the broad prevalence of *SLA-1* duplication from the early period of pig speciation^28,32^. The distant phylogenetic relationships between the two alleles belonging to the same haplotype is also consistent with the history of *SLA-1* duplication in pigs (Table 3, Fig. S3).

*SLA-5* and *SLA-12* were also duplicated among the *SLA* genes but they remain nonfunctional due to the presence of the premature stop codons in their coding region^36^. In contrast, multiple copies of *SLA-1* which are functional could serve as a unique system to increase the potential for presenting diverse peptides to cytotoxic T cells.

The higher frequency of *SLA-1* duplication in the miniature pigs used in biomedical studies, such as SNU (100%) and NIH (76%), than in other pig breeds, was observed. It is interesting to note that similar results were reported for the Sinclair and Hanford breeds^37^.

Several inbred lines were selected as homozygous for SLA in NIH miniature pigs for biomedical studies^38^. Previous and current studies showed the establishment of homozygosity in class II (observed heterozygosity, *DRB1*-0%; *DQA1*-0%; *DQB1*-34.72%) but not in class I genes (observed heterozygosity, *SLA-1*, 83% and *SLA-2*, 46.6%)^18,20–22^ for the breed. Our analysis on *SLA-1* diversity in NIH miniature pigs, however, showed that the high *SLA-1* (83%) heterozygosity was not due to the actual genetic diversity at the chromosomal level but to the presence of a haplotype with duplicated *SLA-1* (Table 3).

In general, the increase of genetic diversity could benefit organismal fitness and disease resistance^39^. However, studies also suggested that there might be trade-offs between MHC variations and immune capacity by T-cell, deletion and reduced immunocompetence, dominant MHC susceptibility alleles to infectious and autoimmune diseases, MHC cell-surface concentration, and T-cell activation^33,40^.

The high prevalence of *SLA-1* duplication makes it difficult to determine the allelic constitutions of *SLA-1* in pigs. Here we observed *SLA-1* duplication from a large number of animals (n = 123) and were able to analyze their haplotypes according to our haplotype phasing strategy (Fig. S5). Our results from the analysis of pig cell lines showed that both CNVs and allelic variations may contribute to the level of *SLA-1* expression (Fig. 4). However, further studies are necessary to address the underlying mechanism for the allelic difference in *SLA-1* expression.

Classical class I *MHC* molecules need to be associated with beta 2 microglobulin (B2M) to become functional. Interestingly, *B2M* was also duplicated in pigs^41^. Thus, dosage increase in one of the heavy chains to form *MHC* class I complexes balances with the duplication of the light chain, B2M.

The reported *SLA-1* typing method in this study requires a single PCR and two sequencing reactions. The procedure for allele discrimination based on local BLAST is simple if the allele database is well prepared. In our method, the most laborious step is the characterization of new alleles that requires cloning and additional sequencing. With further improvements in the list of characterized *SLA-1* alleles and duplicated haplotypes, our method can be applicable to high throughput typing.

To infer the complete *SLA* haplotypes including both class I and II genes from diverse pig breeds could deepen our understanding on highly polymorphic MHC genes in pigs and may reveal the importance of their variations to adaptive immune response. Although genomic DNA-based high-resolution typing is currently available for major *SLA* genes except *SLA-3*, simultaneous typing of major *SLA* genes with high accuracy against a large number of animals is still challenging.

As the cost of next generation sequencing decreases, several recent reports addressed the simultaneous capturing of multiple *MHC* loci in humans^42–44^ and other species including pigs^32^. However, this approach requires both the precise computational procedures to assemble short read sequences and sufficient preceded information on the allelic diversity of *MHC* genes to avoid errors in haplotype phasing and allelic designation^45,46^. Therefore, our results could serve as the basis for the high throughput analysis of the SLA system in the future.

## Conclusions

We developed a comprehensive high-resolution typing of *SLA-1* using single-genomic PCR and subsequent direct sequencing, and reported the identification of new alleles and duplication haplotypes from the typing of over 300 animals from diverse breeds and cell lines. There was a significant difference in the distribution of *SLA-1* alleles among the pig breeds including breed-specific alleles. We presented the results of the population level analysis of *SLA-1* duplication, for the first time. The high frequency of *SLA-1* duplication was observed in the miniature pig breeds use in biomedical studies. We also showed that the duplication of *SLA-1* results in an increase of *SLA-1* expression in pig cells. In our opinion, this is the most comprehensive and large scale study of *SLA-1*.

## Materials and Methods

### Animals and cells

Experiments were conducted using DNA from a total of 295 pigs from 14 different breeds or genetic backgrounds, including 114 Korean native pigs (KNP), 52 Seoul National University (SNU) miniature pig originated from Minnesota miniature pigs^47^, 29 NIH miniature pigs, 21 Duroc, 19 Berkshire, 19 Yorkshire, 19 Landrace, 6 Landrace x KNP cross, 5 Lanyu, 5 Ossabaw Island hog, 4 Meishan, and 2 American Guinea Hog (AGH). Among the animals used in this study, 48 had pedigree information (Supplementary Table S2). In addition, 8 pig cell lines (CCL-166, CRL-1746, CL-184, CRL-2528, CRL-2842, CRL-6489, CL-101, and CCL-33) from the American Type Culture Collection (ATCC, Manassas, VA, USA) and 4 primary porcine alveolar macrophages (PAM) cells were also analyzed for validation of the typing results. ATCC fibroblast cell lines were cultured in DMEM/high glucose media (Hyclone, UT, USA) supplemented with 10% FBS (Hyclone), 1% penicillin-streptomycin (Gibco, NY, USA), and 2 mM L-glutamine (Gibco) with 5% CO_2_ and 37 °C. PAMs were isolated from ten 10-week-old euthanized healthy Yorkshire x Landrace cross pigs raised at a local farm using bronchoalveolar lavage through a conventional method^48^. All experiments were approved and performed accordance with the guidelines and regulations set by Institute of Animal Care and Use Committee and the Center for Research Ethics of Konkuk University

### Specific amplification of SLA-1 from genomic DNA

The preparation of genomic DNA and mRNA is described as Supplementary Information. To determine *SLA-1* specific primer binding sites, 2086 bp amplicons corresponding to the region from 5′ UTR to the middle of exon 4 of *SLA class I* genes were produced by PCR with 0.5 μM *SLA* class I specific primers (SLA-CATF and SLA-R, Table 1) as described previously^21^. For genomic DNA-based typing, *SLA-1* specific amplicons were generated in a 20-μL reaction containing 50–100 ng of genomic DNA, 0.5 μM specific primers (SLA1-e1F1 and SLA e4R4) (Table 1), 200 μM dNTPs, 1 × PCR buffer, and 0.5 U of Supertherm™ DNA polymerase (JMR Holdings, Kent, UK) using a ABI 9700 Thermocycler (Applied Biosystems). The cycling profile consisted of an initial denaturation at 94 °C for 5 min, followed by 35 cycles of denaturation at 94 °C for 1 min, annealing at 65 °C for 1 min, and extension at 72 °C for 2 min, and a final extension at 72 °C for 10 min. The PCR products were checked by electrophoresis on a 1.5% agarose gel.

### Specific amplification of SLA-1 from cDNA

Reverse transcription was carried out according to the manufacturer’s instructions in a 20-μL reaction with oligo(dT)15 and SuperScript™ III reverse transcriptase (Invitrogen, Carlsbad, CA) as described previously^21^. Two microliters of cDNA were used to amplify the *SLA-1* transcripts using SLA1-e1F1 and SLA-e4R4 primers (Table 1) with a cycling profile of an initial denaturation at 94 °C for 5 min, followed by 35 cycles of denaturation at 94 °C for 1 min, annealing at 65 °C for 1 min, and extension at 72 °C for 1 min, and a final extension at 72 °C for 10 min.

### DNA sequencing for SLA-1 typing

The preparation of templates for direct sequencing of PCR products was as described previously^18^. Sequencing reactions were performed using the ABI PRISM BigDye™ Terminator Cycle Sequencing Kit (Applied Biosystems, Foster City, CA) with the sequencing primers SLA1-Seq. 2-F, SLA1-Seq. 2-R, SLA1-Seq. 3-F, and SLA1-Seq. 3-R (Table 1). The RT-PCR products cloned in the PCR-Script Amp SK( + ) cloning vector were bidirectionally sequenced using the T3 and T7 universal sequencing primers. Bidirectional sequencing of the cloned inserts within the pGEM-T Easy Vector for new allele confirmation was carried out using the T7 and SP6 universal primers. The procedures for plasmid sequencing was the same as those of direct sequencing except that the step for removing the unincorporated primers was excluded. A total of 8~10 clones were sequenced bidirectionally for each ligation to eliminate possible sequencing artifacts. For the samples containing new alleles, independent PCR products were sequenced at least twice.

### Allelic discrimination of SLA-1

The *SLA-1* alleles were discriminated using nucleotide sequence alignment as described previously^21^. Briefly, chromatograms with sequence quality values >20 were used for the allelic designation of the homozygotes. To separate and identify each allele in the heterozygotes, the PCR products were cloned when the pattern was observed for the first time, and the individual clones of each allele were sequenced. Since a single sequencing template contains information of both exons 2 and 3, the exonal combination was not an issue in our typing method. The sequencing results from 5ʹ and 3ʹ directions were imported into the CLC main workbench version 7.8.1 (CLC Bio, Aarhus, Denmark) for assembly. The resulting 546-bp sequence containing both *SLA-1* exon 2 and exon 3 was used in a BLAST search against existing alleles in the local SLA database, which contains information of all reported alleles in public databases and new alleles from our *SLA-1* typing. The best five matches were selected and aligned to the query sequence. The allele with any discrepancy compared to the query sequence was removed reiteratively and the final filtered alignment will contain the allele set of the typed sample. The sequence information of the new alleles was submitted to Genbank and the official allele names were designated by the International Society of Animal Genetics (ISAG) SLA nomenclature committee under established guidelines^49^.

### Determination of SLA-1 duplication and haplotypes

The typing results of more than 2 alleles indicates the presence of two copies of *SLA-1* alleles on the same chromosome caused by *SLA-1* duplication. The determination of genetic linkage between alleles of duplicated *SLA-1* are necessary for genetic analyses. The putative haplotypes for duplicated *SLA-1* loci was established when the specific allele combination of previously reported^24,28^ was observed or a specific allele combination was always observed together in the typing results, and the strategy was described in Fig. S5. Subsequently, the identified haplotypes of *SLA-1* duplication were applied to deduce allelic combinations of the typing results.

### Population genetics analysis

Because of the *SLA-1* duplication, the determination of genetic parameters including allele frequency and observed heterozygosity differs from that of a physically single locus. In our analysis, we calculated the values from typing results consisting of all detected *SLA-1* in a given individual regardless of the copy numbers of *SLA-1*. Therefore, the heterozygosity indicates the presence of more than one allele regardless of the duplication. The expected heterozygosity was calculated according to Hartl *et al*.^50^. The pairwise Nei’s genetic identity^51^, Shannon’s diversity index, and allelic evenness^52^, were calculated as previously reported on the basis of the determined allele frequencies, which was calculated by dividing the total number of the observed allele with the total number of alleles while considering the number of involved *SLA-1* loci from the duplication (Table S1). The breed specificity of *SLA-1* alleles was visualized using the Jvenn software (http://bioinfo.genotoul.fr/jvenn/index.html). Phylogenetic analysis was performed using the Neighbor Joining method under the general time reversible model as the best fit model in CLC main workbench (CLC Bio).

### Quantification of SLA-1 expression using real-time PCR

cDNA was prepared as described above. Primers, SLA1-e1F1 and SLA-e12R, were designed for the specific amplification (137 bp) of *SLA-1* transcripts (Table 1). SLA-e12R was located at the junction of *SLA-1* exons 1 and 2 to avoid amplification from the genomic DNA (Table 1, Fig. 2). Four ATCC pig cell lines with confirmed *SLA-1* types, CL-184 and CRL-17462 with two copies, and CCL-166 and CRL-2528 with 3 copies were used as controls^29^ (Table S3). Real-time PCR was carried out in a 25-μL reaction containing 1 μL of synthesized cDNA and 0.25 μM of each primer in a 1 × solution of SsoAdvanced™ Universal SYBR® Green Supermix using CFX ConnectTM RealTime System (Bio-Rad, CA, US). The cycling condition of the two-step amplification consisted of an initial denaturation at 94 °C for 3 min; 40 cycles of denaturation at 94 °C for 15 s; annealing for 30 s at 65 °C for *SLA-1* and 53 °C for *GAPDH*; followed by melting curve analysis from 65 °C to 95 °C with an increment of 0.5 °C in step of 0.05 s. The reaction was repeated three times for each sample. The PCR results were analyzed by Bio-Rad CFX Manager, version 3.1 (Bio-Rad). The 2^−ΔΔCt^ method was used for the relative quantification to determine the relative expression level. *GAPDH* was used as the control gene and was amplified as a 123-bp amplicon using the primers GAPDH-F2 and GAPDH-R2 (Table 1). Statistical analysis was conducted using the Student’s *t*-test.

## Supplementary information

Supplementary information.
