# Supplementary material for: SLA-1 Genetic Diversity in Pigs: Extensive Analysis of Copy Number Variation, Heterozygosity, Expression, and Breed Specificity
Source: Sci Rep. 2020 Jan 20;10:743. doi: 10.1038/s41598-020-57712-5 (PMC6971002; doi:10.1038/s41598-020-57712-5)
Supplement: Supplementary file 1 — Supplementary information. [file 41598_2020_57712_MOESM1_ESM.pdf]

Supplementary Information

SLA-1 Genetic Diversity in Pigs: Extensive Analysis of Copy Number Variation, Heterozygosity, Expression, and Breed Specificity

Minh Thong Le[1,3], Hojun Choi[1], Hyejeong Lee[1], Van Chanh Quy Le[1], Byeongyong Ahn[1], Chak-Sum Ho[2], Kwonho Hong[1], Hyuk Song[1], Jin-Hoi Kim[1], Chankyu Park[1,*]

[1]*Department of Stem Cells and Regenerative Biology, Konkuk University, Seoul 143-701, Korea*
[2]*Gift of Life Michigan, Ann Arbor, MI, 48108, USA*
[3]**Current address:**
School of Biotechnology, International University - Vietnam National University HCMC, Ho Chi Minh 700000, Vietnam

[*]Corresponding author:
Chankyu Park,
Department of Stem Cells and Regenerative Biology, Konkuk University,
Hwayang-dong, Kwangjin-gu,
Seoul 143-701, Korea

Tel: 82-2-450-3697; Fax: 82-2-457-8488

E-mail: chankyu@konkuk.ac.kr

Materials and methods:

Preparation of genomic DNA and mRNA

Genomic DNA was prepared from ~0.5 g of tissues obtained from ear punch or 1 mL of blood containing 6% ethylenediaminetetraacetic acid (EDTA) as previously described (Choi et. al., 2015). A total of 5×106 trypsinized cells were incubated with lysis buffer [10 mM Tris-HCl (pH 8.0), 0.1 M EDTA, 0.5% sodium dodecyl sulphate (SDS), and 1 mg/mL proteinase K (Promega, Madison, WI)] at 55 oC for 4 h. The remainder of the procedures followed were the same as that of DNA isolation from tissues. The mRNA was extracted from the cells using the Trizol reagent (Invitrogen, CA, USA) and purified using RNeasy™ Mini kits (Qiagen, Hilden, Germany) according to the manufacturer's instructions. DNA concentration and integrity were checked by electrophoresis on 1% agarose gel in 1× tris-acetate-EDTA (TAE) buffer. The gel was stained with ethidium bromide and visualized under UV light.

DNA cloning and plasmid isolation

The products of *SLA class I* gene-specific PCRs were gel-purified using QIAquick Gel Extraction kit (Qiagen, Hamburg, Germany) and cloned into the PCR-Script Amp SK(+) (Promega, WI, USA) or pGEM-T Easy cloning vector (Stratagene, La Jolla, CA). The ligation products were electroporated into DH5α cells using a MicroPulser (Biorad, Hercules, CA) and the transformed bacteria were plated onto agar containing 50 µg/mL ampicillin, 40 µg/mL X-gal solution, and 0.1 mM isopropyl-β-D-1-thiogalctopyranoside (IPTG). The plasmids were isolated using Plasmid SV Miniprep kit (GeneAll Biotechnology, Seoul, Korea).

Figure S1. The results of PCR amplification using the *SLA-1*-specific primer pair, SLA1-e1F1 and SLA-e4R4, against 7 breeds and 2 cell lines. The 1844-bp segment of the SLA-DQB1–specific band was consistently amplified from all samples. Sample names are indicated at the top. M, DNA marker.

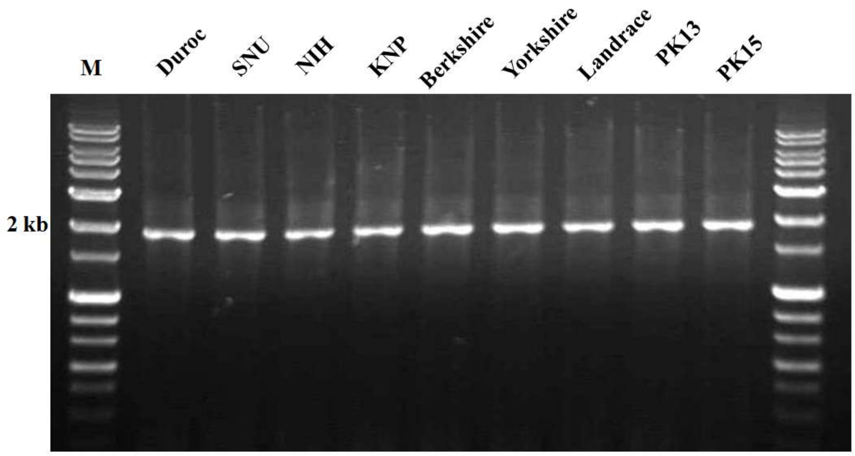

Figure S2: Multiple alignment of the 9 *SLA-1* alleles containing a region from intron 1 to exon 4

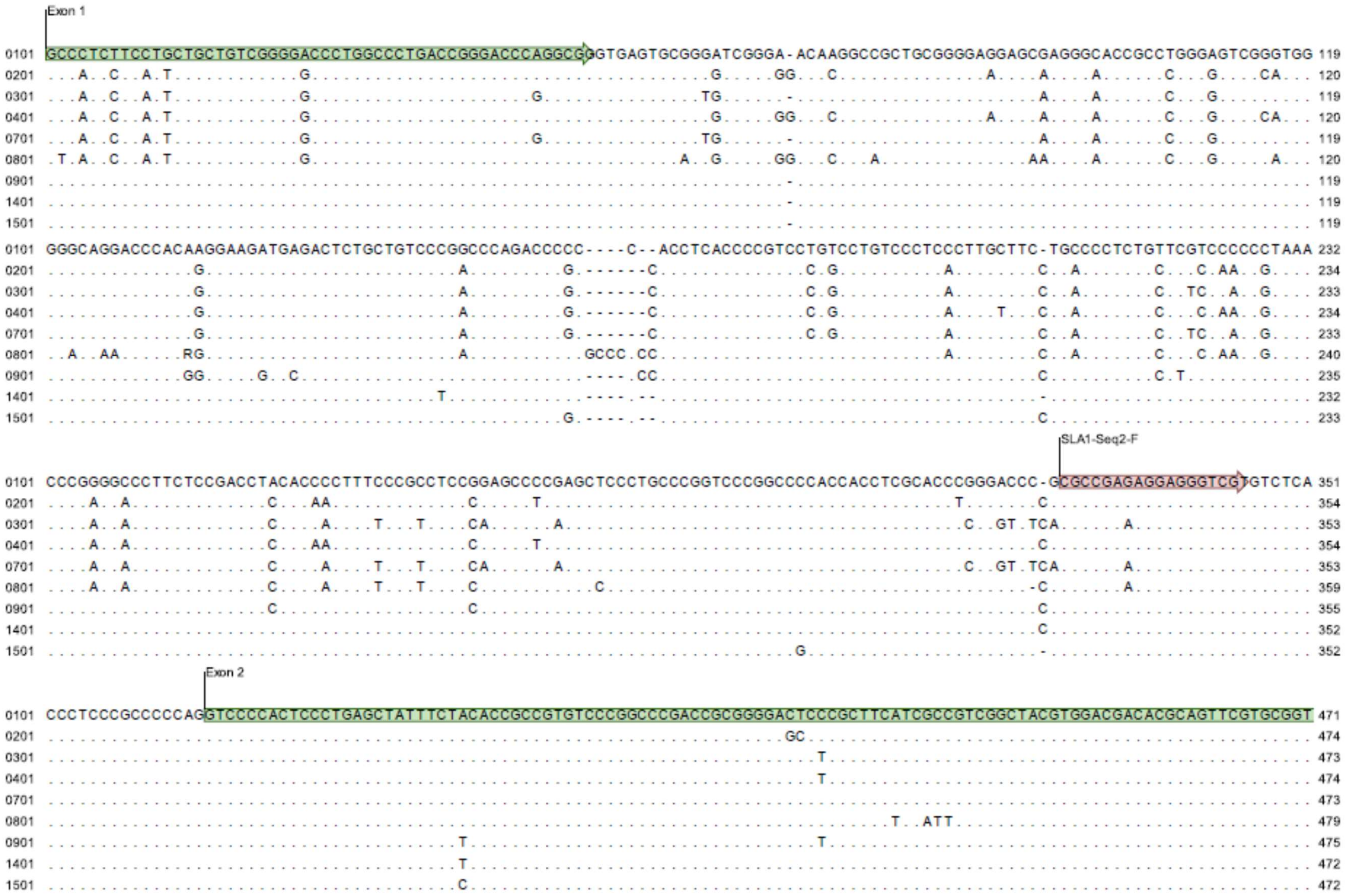

Figure S2: (cont)

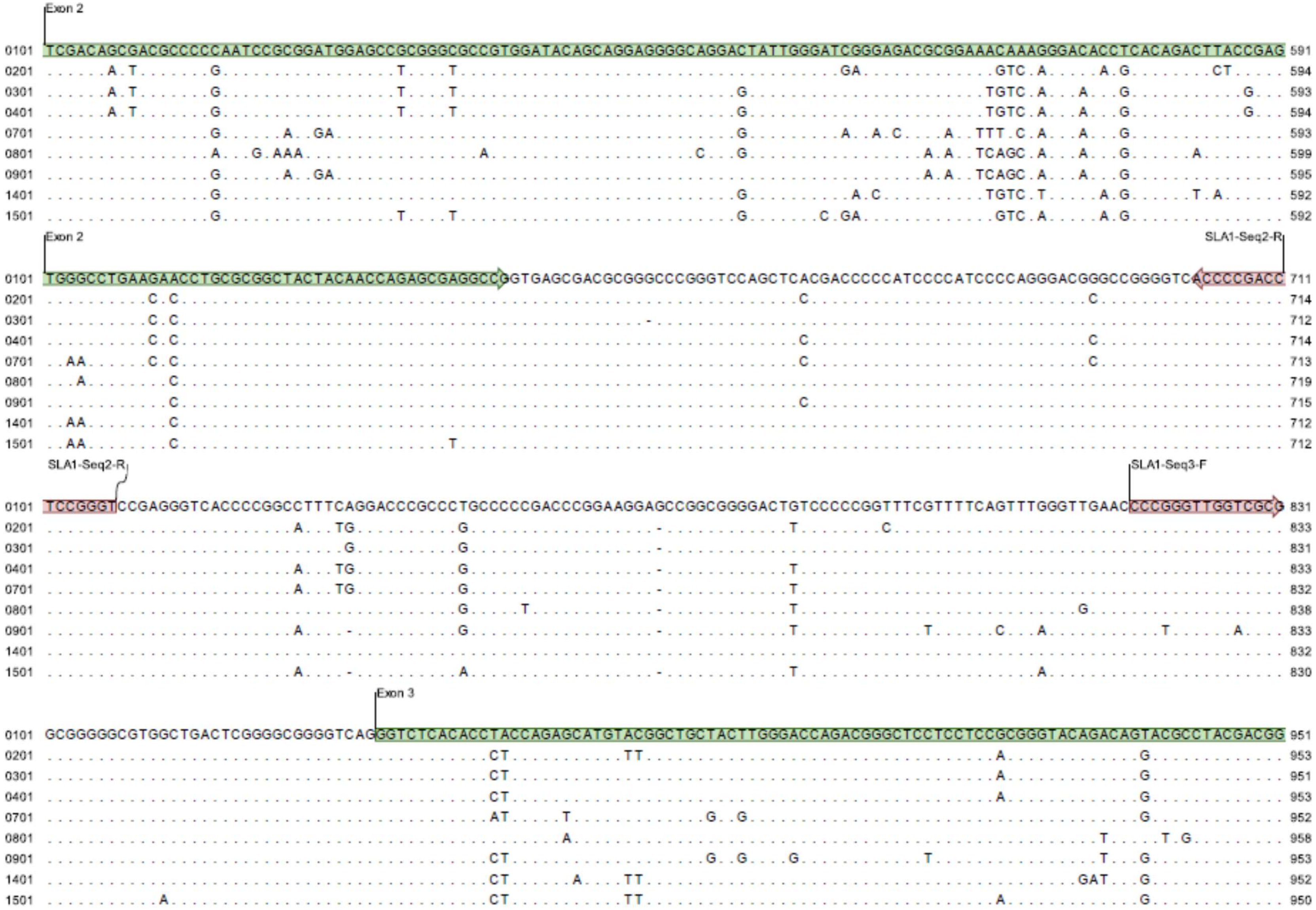

Figure S2: (cont)

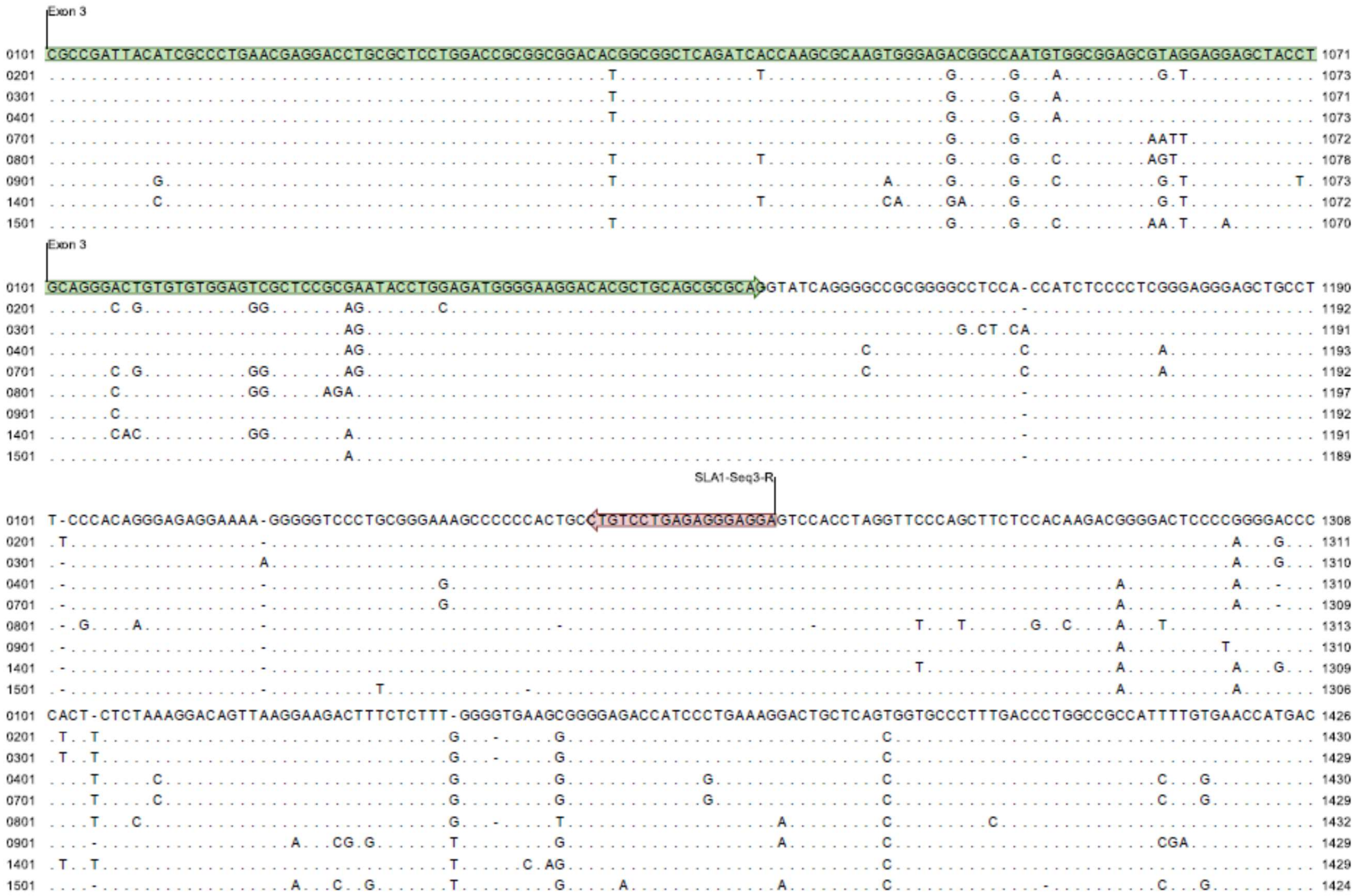

Figure S2: (cont)

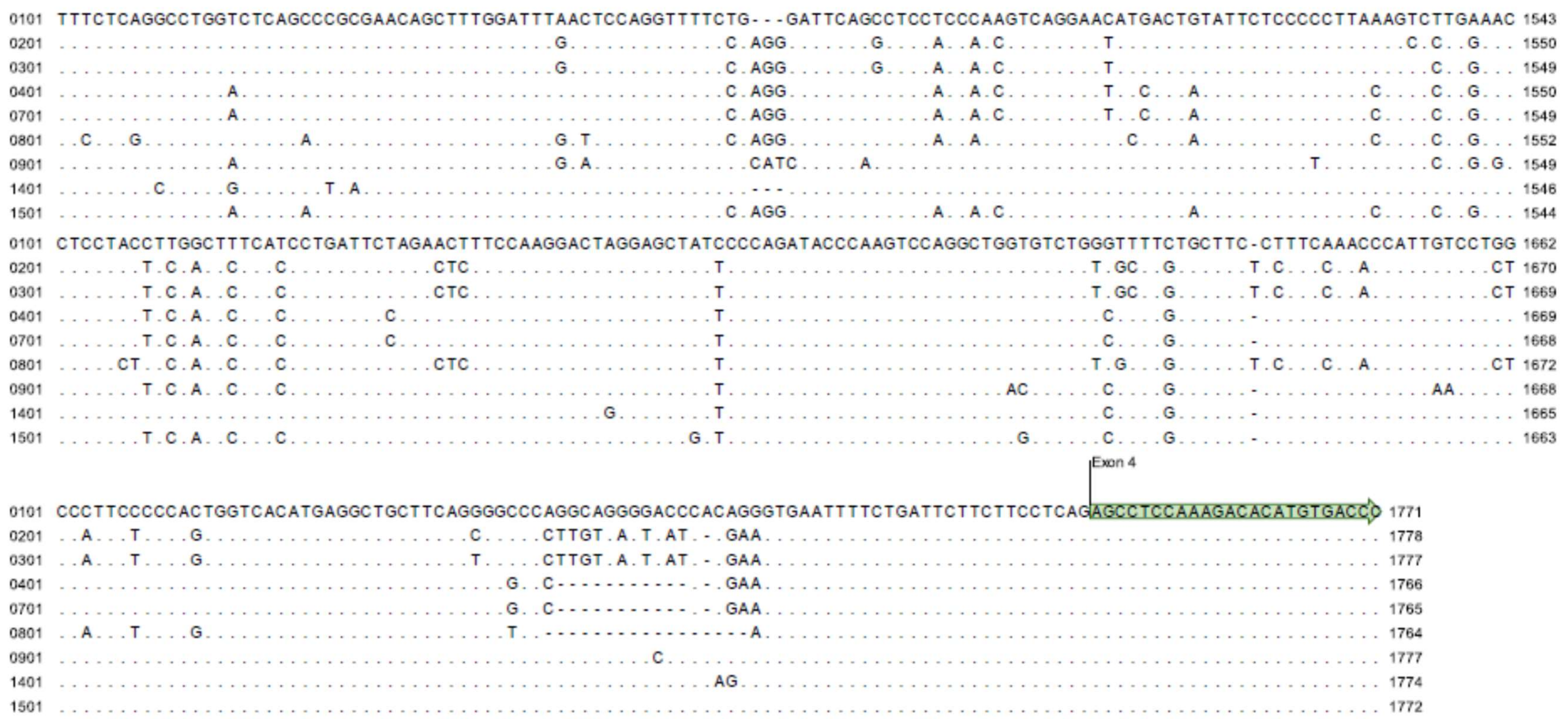

Figure S3. The results of a phylogenetic analysis using 69 *SLA-1* alleles present in the IPD database and 18 new alleles with the complete exon 2 and 3 sequence information. HLA-A*02, -B*07, -C*07 were used as out group. The numbers on the nodes indicate the bootstrap values (n = 100). Alleles with '*', at the ends of their names indicate that the alleles were shown in our genotyping results.

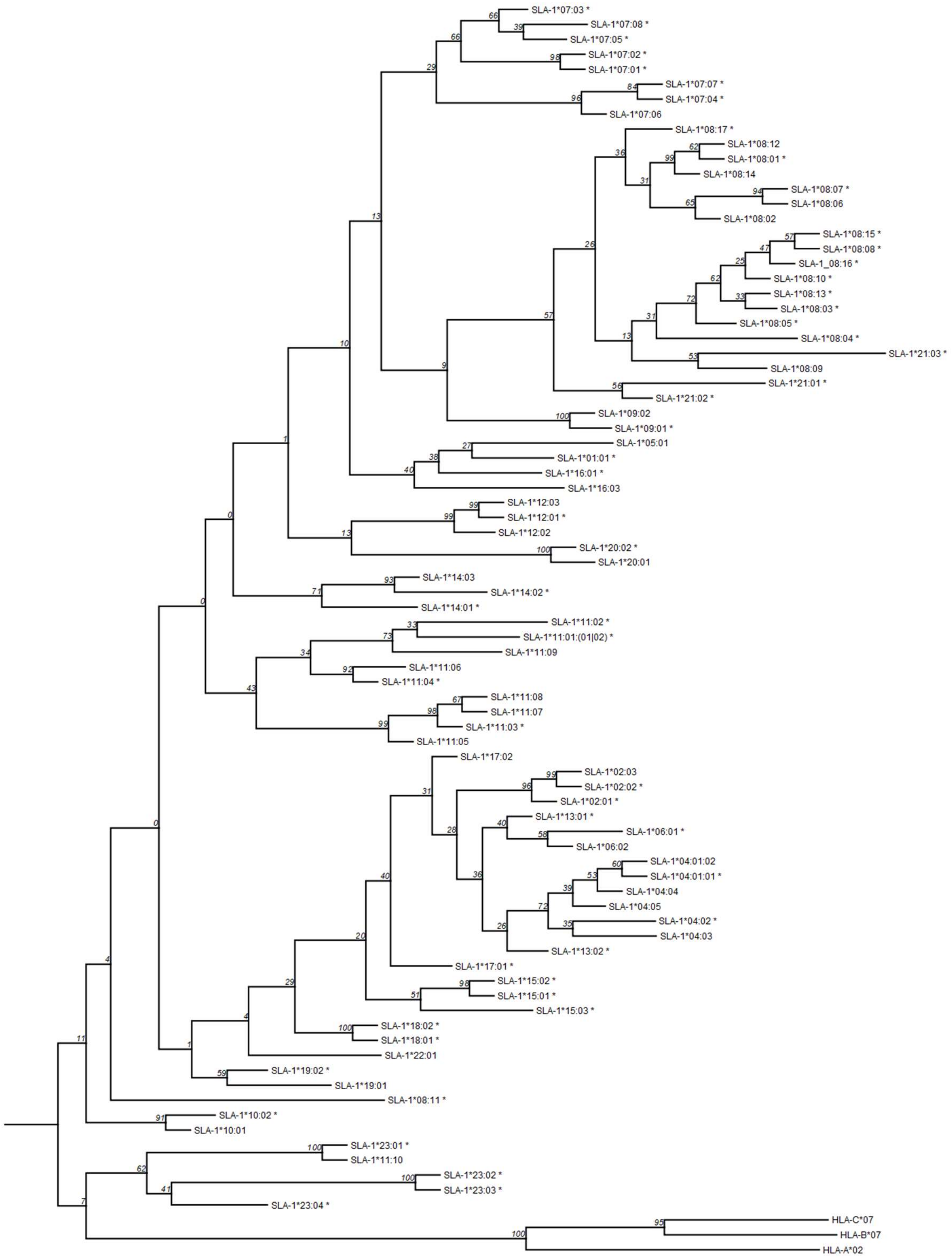

Figure S4. The distribution of breed specific and shared *SLA-1* alleles. The numbers of breed-shared and -specific alleles are indicated inside the Venn diagram (**A**). The bar chart in the middle indicates the total number of observed alleles in each breed (**B**). The numbers of alleles shared with the number of breeds are shown inside and below the bars, respectively (**C**). The details are summarized in Supplementary Table S6.

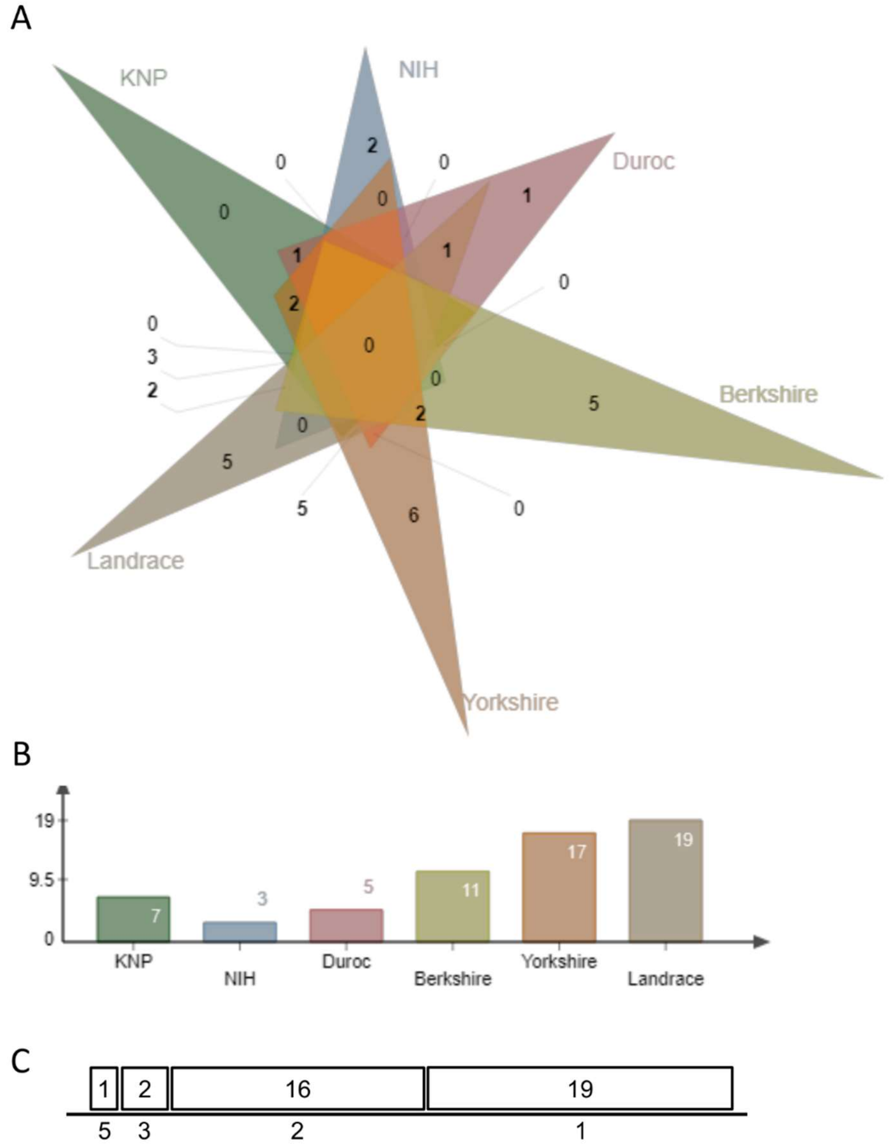

Figure S5. The strategy for the determination of duplication haplotype of *SLA-1.* A. The diagram shows the steps of haplotype determination. B. The diagram describes the definition of duplication homozygote, heterozygote and hemizygote. The letters "a", "b", "c"," d", and "x" indicate allele names. The same latter and "x" indicate the same and any allele, respectively.

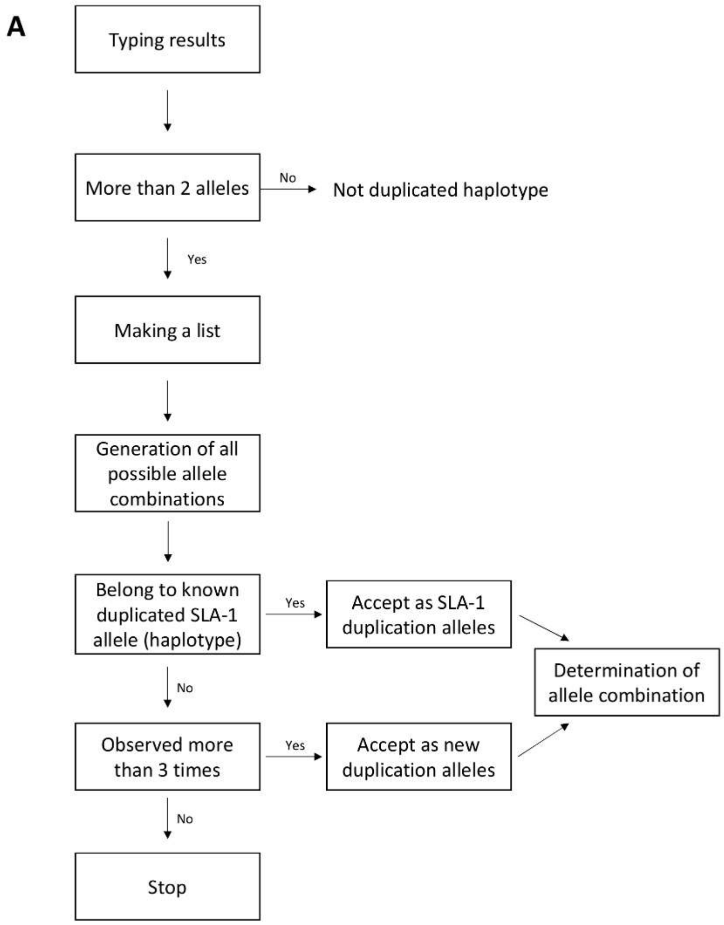

Table S1. Results of *SLA-1* typing for entire samples in this study.

Sample names		*SLA-1* typing results	Haplotype phases	Number of copies
Berkshire	B01	23:02/21:02	23:02-21:02//23:02-21:02	4
	B02	15:03/23:02/21:02	15:03//23:02-21:02	3
	B03	08:15/23:02/21:02	08:15//23:02-21:02	3
	B04	08:15/23:02/21:02	08:15//23:02-21:02	3
	B05	15:03/23:02/21:02	15:03//23:02-21:02	3
	B06	11:01/23:02/21:02	11:01//23:02-21:02	3
	B07	08:15/04:02/23:04	04:02//08:15//23:04	3
	B08	21:02/23:02	23:02-21:02//23:02-21:02	4
	B09	08:15/23:04	08:15//23:04	2
	B10	11:01	11:01//11:01	2
	B11	04:01/15:03	04:01//15:03	2
	B12	11:04/15:03	11:04//15:03	2
	B13	23:04/08:15/15:03	08:15//23:04//15:03	3
	B14	23:02/23:04/21:02	23:02-21:02//23:04	3
	B15	11:04/23:04/08:15	11:04//08:15//23:04	3
	B16	11:04	11:04//11:04	2
	B17	21:02/23:02/23:04	23:02-21:02//23:04	3
	B18	04:02/23:02/21:02	23:02-21:02//04:02	3
	B19	07:02/14:01	07:02//14:01	2
Duroc	D01	04:01	04:01//04:01	2
	D02	07:02/11:02	07:02//11:02	2
	D03	04:01/08:17	04:01//08:17	2
	D04	04:01	04:01//04:01	2
	D05	11:02/08:17	11:02//08:17	2
	D06	04:01/08:17	04:01//08:17	2
	D07	08:17	08:17//08:17	2
	D08	04:01/08:17	04:01//08:17	2

	D09	04:01	04:01//04:01	2
	D10	04:01	04:01//04:01	2
	D11	04:01	04:01//04:01	2
	D12	04:01	04:01//04:01	2
	D13	04:01/16:01	04:01//16:01	2
	D14	04:01	04:01//04:01	2
	D15	04:01	04:01//04:01	2
	D16	04:01	04:01//04:01	2
	D17	04:01/08:17	04:01//08:17	2
	D18	04:01	04:01//04:01	2
	D19	04:01	04:01//04:01	2
	D20	04:01	04:01//04:01	2
	D21	04:01	04:01//04:01	2
Yorkshire	Y01	08:11/21:03	08:11//21:03	2
	Y02	04:01/07:08	04:01//07:08	2
	Y03	02:02/08:08/18:01	02:02-18:01//08:08	3
	Y04	13:01/07:03/12:01	12:01-13:01//07:03	3
	Y05	11:03/21:01/08:10	11:03-21:01//08:10	3
	Y06	02:02/11:03/18:01/21:01	02:02-18:01//11:03-21:01	4
	Y07	02:02/08:08/18:01	02:02-18:01//08:08	3
	Y08	08:10	08:10//08:10	2
	Y09	08:08/11:03/21:03	11:03//21:03//08:08	3
	Y10	02:02/18:01/11:03/21:01	02:02-18:01//11:03-21:01	4
	Y11	01:01/07:04	01:01//07:04	2
	Y12	01:01/08:10	01:01//08:10	2
	Y13	08:10	08:10//08:10	2
	Y14	01:01/08:10	01:01//08:10	2
	Y15	01:01/07:08	01:01//07:08	2
	Y16	04:01/07:08	04:01//07:08	2
	Y17	11:01/23:04/08:08	11:01//08:08//23:04	3
	Y18	01:01/08:10	01:01//08:10	2

	Y19	12:01/13:01/07:08	12:01-13:01//07:08	3
SNU	S1	02:01/07:01	02:01-07:01//02:01-07:01	4
	S2	02:01/07:01	02:01-07:01//02:01-07:01	4
	S3	02:01/07:01	02:01-07:01//02:01-07:01	4
	S4	02:01/07:01	02:01-07:01//02:01-07:01	4
	S5	02:01/07:01	02:01-07:01//02:01-07:01	4
	S6	02:01/07:01	02:01-07:01//02:01-07:01	4
	S7	02:01/07:01	02:01-07:01//02:01-07:01	4
	S8	02:01/07:01	02:01-07:01//02:01-07:01	4
	S9	02:01/07:01	02:01-07:01//02:01-07:01	4
	S10	02:01/07:01	02:01-07:01//02:01-07:01	4
	S11	06:01/18:02	0601-18:02//0601-18:02	4
	S17	02:01/07:01	02:01-07:01//02:01-07:01	4
	S19	02:01/07:01	02:01-07:01//02:01-07:01	4
	S22	02:01/07:01	02:01-07:01//02:01-07:01	4
	S27	02:01/07:01	02:01-07:01//02:01-07:01	4
	S28	02:01/07:01	02:01-07:01//02:01-07:01	4
	S30	02:01/07:01	02:01-07:01//02:01-07:01	4
	S33	06:01/18:02	0601-18:02//0601-18:02	4
	S36	06:01/18:02	0601-18:02//0601-18:02	4
	S38	02:01/07:01	02:01-07:01//02:01-07:01	4
	S40	02:01/07:01	02:01-07:01//02:01-07:01	4
	S41	06:01/18:02	0601-18:02//0601-18:02	4
	S48	02:01/07:01	02:01-07:01//02:01-07:01	4
	S51	02:01/07:01	02:01-07:01//02:01-07:01	4
	S52	02:01/07:01	02:01-07:01//02:01-07:01	4
	S53	02:01/07:01	02:01-07:01//02:01-07:01	4
	S54	02:01/07:01	02:01-07:01//02:01-07:01	4
	S56	02:01/07:01	02:01-07:01//02:01-07:01	4
	S58	02:01/07:01	02:01-07:01//02:01-07:01	4
	S59	06:01/18:02	06:01-18:02//0601-18:02	4

	S63	02:01/07:01	02:01-07:01//02:01-07:01	4
	S64	02:01/07:01	02:01-07:01//02:01-07:01	4
	S71	02:01/07:01	02:01-07:01//02:01-07:01	4
	S76	06:01/18:02	0601-18:02//0601-18:02	4
	S79	02:01/07:01	02:01-07:01//02:01-07:01	4
	S93	02:01/06:01/07:01/18:02	02:01-07:01//0601-18:02	4
	S95	02:01/06:01/07:01/18:02	02:01-07:01//0601-18:02	4
	S110	02:01/07:01	02:01-07:01//02:01-07:01	4
	S61	02:01/07:01	02:01-07:01//02:01-07:01	4
	CMS1	02:01/07:01	02:01-07:01//02:01-07:01	4
	CMS2	02:01/07:01	02:01-07:01//02:01-07:01	4
	CMS5	02:01/07:01	02:01-07:01//02:01-07:01	4
	CMS6	02:01/07:01	02:01-07:01//02:01-07:01	4
	CMS8	02:01/07:01	02:01-07:01//02:01-07:01	4
	CMS9	02:01/07:01	02:01-07:01//02:01-07:01	4
	CMS10	02:01/07:01	02:01-07:01//02:01-07:01	4
	CMS11	02:01/07:01	02:01-07:01//02:01-07:01	4
	CMS12	02:01/07:01	02:01-07:01//02:01-07:01	4
	CMS13	02:01/07:01	02:01-07:01//02:01-07:01	4
	CMS15	02:01/07:01	02:01-07:01//02:01-07:01	4
	CMS16	02:01/07:01	02:01-07:01//02:01-07:01	4
	CMS20	02:01/07:01	02:01-07:01//02:01-07:01	4
KNP	J105	08:01/11:02	08:01//11:02	2
	J16-33	11:02/11:03/21:01	11:02//11:03-21:01	3
	J2001	08:01/12:01	08:01//12:01	2
	J2004	08:01	08:01//08:01	2
	J2008	08:01/11:02	08:01//11:02	2
	J2013	11:02	11:02//11:02	2
	J2013	11:02/12:01	11:02//12:01	2
	J2013	11:02/12:01	11:02//12:01	2
	J2022	08:01/11:03/21:01	11:03-21:01//08:01	3

J2029	08:01	08:01//08:01	2
J2031	08:01/11:02	08:01//11:02	2
J2032	11:03/15:02/21:01	11:03-21:01//15:02	3
J2035	11:02/15:02	11:02//15:02	2
J2036	11:02	11:02//11:02	2
J2038	11:02/15:02	11:02//15:02	2
J2041	08:01/11:02	08:01//11:02	2
J2044	08:01	08:01//08:01	2
J2047	08:01	08:01//08:01	2
J2049	08:01	08:01//08:01	2
J2050	08:01	08:01//08:01	2
J3001	08:01	08:01//08:01	2
J3003	08:01/11:03/21:01	11:03-21:01//08:01	3
J3004	08:01/11:03/21:01	11:03-21:01//08:01	3
J3006	08:01	08:01//08:01	2
J3007	08:01	08:01//08:01	2
J3008	11:02/12:01	11:02//12:01	2
J3012	11:02/15:02	11:02//15:02	2
J3013	15:02	15:02//15:02	2
J3014	11:02/15:02	11:02//15:02	2
J3015	15:02	15:02//15:02	2
J3016	11:02	11:02//11:02	2
J3017	11:02/15:02	11:02//15:02	2
J3018	08:01/11:02	08:01//11:02	2
J3019	08:01/11:02	08:01//11:02	2
J3020	11:02/15:02	11:02//15:02	2
J3021	11:02	11:02//11:02	2
J3022	08:01/11:02	08:01//11:02	2
J3023	11:02/12:01	11:02//12:01	2
J3024	11:02/12:01	11:02//12:01	2
J3025	08:01	08:01//08:01	2

J3026	08:01/11:02	08:01//11:02	2
J3027	11:02/12:01	11:02//12:01	2
J3028	11:02/12:01	11:02//12:01	2
J3029	08:01/11:02	08:01//11:02	2
J3030	08:01/11:02	08:01//11:02	2
J3031	08:01/11:02	08:01//11:02	2
J3032	11:02	11:02//11:02	2
J3034	11:02	11:02//11:02	2
J4003	08:01/11:02	08:01//11:02	2
J4004	08:01/11:02	08:01//11:02	2
J4006	08:01/11:02	08:01//11:02	2
J4007	11:02	11:02//11:02	2
J4008	11:02/15:02	11:02//15:02	2
J4009	08:01/12:01	08:01//12:01	2
J4012	15:02	15:02//15:02	2
J4013	11:02/12:01	11:02//12:01	2
J4014	11:02/15:02	11:02//15:02	2
J4015	15:02	15:02//15:02	2
J4017	11:02/15:01	11:02//15:01	2
J4018	11:02/15:01	11:02//15:01	2
J4023	11:02	11:02//11:02	2
J4024	11:02	11:02//11:02	2
j4026	11:02/15:01	11:02//15:01	2
J4027	12:01	12:01//12:01	2
J4029	08:01/11:03/21:01	08:01//11:03-21:01	3
J5001	08:01/11:02	08:01//11:02	2
J5002	08:01	08:01//08:01	2
K01	15:02	15:02//15:02	2
K06	08:01/12:01	08:01//12:01	2
K08	11:02/12:01	11:02//12:01	2
K11	08:01/11:02	08:01//11:02	2

K12	08:01/11:02	08:01//11:02	2
K13	11:02	11:02//11:02	2
K14	08:01/11:02	08:01//11:02	2
K15	11:02	11:02//11:02	2
K16	11:02	11:02//11:02	2
K19	08:01	08:01//08:01	2
K20	08:01	08:01//08:01	2
K22	08:01	08:01//08:01	2
K27	11:02/15:02	11:02//15:02	2
K28	8:01	08:01//08:01	2
K29	08:01/11:02	08:01//11:02	2
K30	15:02	15:02//15:02	2
K31	11:02	11:02//11:02	2
K32	11:02	11:02//11:02	2
K34	11:03/15:02/21:01	11:03-21:01//15:02	3
K35	11:02	11:02//11:02	2
KNP1	11:02/15:02	11:02/15:02	2
KNP10	11:02/12:01	11:02//12:01	2
KNP11	08:01	08:01//08:01	2
KNP12	08:01/15:02	08:01//15:02	2
KNP13	08:01/15:02	08:01//15:02	2
KNP14	08:01/11:02	08:01//11:02	2
KNP15	08:01/11:02	08:01//11:02	2
KNP16	08:01/11:02	08:01//11:02	2
KNP2	11:02/15:02	11:02//15:02	2
KNP3	11:02/15:02	11:02//15:02	2
KNP4	11:02	11:02//11:02	2
KNP5	11:02	11:02//11:02	2
KNP6	11:02	11:02//11:02	2
KNP7	11:02/15:02	11:02//15:02	2
KNP8	11:02/15:02	11:02//15:02	2

	KNP9	11:02/12:01	11:02//12:01	2
	KNP17	08:01/11:02	08:01//11:02	2
	NJ1	08:01/11:02	08:01//11:02	2
	NJ10	08:01/11:02	08:01//11:02	2
	NJ11	11:02/11:03/21:01	11:02//11:03-21:01	3
	NJ2	11:02/11:03/21:01	11:02//11:03-21:01	3
	NJ3	11:02/11:03/21:01	11:02//11:03-21:01	3
	NJ4	08:01/11:02	08:01//11:02	2
	NJ5	11:02/11:03/21:01	11:02//11:03-21:01	3
	NJ6	11:02/11:03/21:01	11:02//11:03-21:01	3
	NJ7	08:01/11:02	08:01//11:02	2
	NJ8	11:02/11:03/21:01	11:02//11:03-21:01	3
Landrace	L01	14:01/15:02	14:01//15:02	2
	L02	14:01/08:01/15:01/08:11	14:01//15:01//08:01//08:11	4
	L03	08:05/08:11	08:05//08:11	2
	L04	14:01/08:17	14:01//08:17	2
	L05	14:01	14:01//14:01	2
	L06	07:02/15:01	07:02//15:01	2
	L07	11:04	11:04//11:04	2
	L08	04:01/21:03	04:01//21:03	2
	L09	08:10/23:01	08:10//23:01	2
	L10	08:03	08:03//08:03	2
	L11	14:01/15:02	14:01//15:02	2
	L12	01:01/12:01/13:01	01:01//12:01-13:01	3
	L13	01:01/14:01	01:01//14:01	2
	L14	04:01/23:01	04:01//23:01	2
	L15	23:01/15:02	23:01//15:02	2
	L16	14:01/20:02	14:01//20:02	2
	L17	23:01/15:01/09:01	15:01-09:01//23:01	3
	L5-68	08:01/15:01/09:01	08:01//15:01-09:01	3
	L5-83	08:11/14:01	08:11//14:01	2

NIH	N1	02:01/07:01	02:01-07:01//02:01-07:01	4
	N2	02:01/07:01	02:01-07:01//02:01-07:01	4
	N3	02:01/07:01	02:01-07:01//02:01-07:01	4
	N4	02:01/07:01	02:01-07:01//02:01-07:01	4
	N5	02:01/07:01	02:01-07:01//02:01-07:01	4
	N6	02:01/07:01	02:01-07:01//02:01-07:01	4
	N7	02:01/07:01	02:01-07:01//02:01-07:01	4
	N8	04:01	04:01//04:01	2
	N9	02:01/07:01	02:01-07:01//02:01-07:01	4
	N10	02:01/07:01	02:01-07:01//02:01-07:01	4
	N11	02:01/04:01/07:01	04:01//02:01-07:01	3
	N12	02:01/07:01	02:01-07:01//02:01-07:01	4
	N13	02:01/04:01/07:01	04:01//02:01-07:01	3
	N14	04:01	04:01//04:01	2
	N15	02:01/04:01/07:01	04:01//02:01-07:01	3
	N16	02:01/07:01	02:01-07:01//02:01-07:01	4
	N17	02:01/07:01	02:01-07:01//02:01-07:01	4
	N18	02:01/07:01	02:01-07:01//02:01-07:01	4
	N19	02:01/07:01	02:01-07:01//02:01-07:01	4
	N20	04:01	04:01//04:01	2
	N21	02:01/07:01	02:01-07:01//02:01-07:01	4
	N22	02:01/07:01	02:01-07:01//02:01-07:01	4
	N23	02:01/07:01	02:01-07:01//02:01-07:01	4
	N24	02:01/07:01	02:01-07:01//02:01-07:01	4
	N25	04:01	04:01//04:01	2
	N26	02:01/04:01/07:01	04:01//02:01-07:01	3
	N27	04:01	04:01//04:01	2
	N28	02:01/07:01	02:01-07:01//02:01-07:01	4
	N29	02:01/07:01	02:01-07:01//02:01-07:01	4
Lanyu	12lu5	04:01/19:02	04:01//19:02	2
	12lu10	04:01	04:01//04:01	2

	12lu16	04:01	04:01//04:01	2
	12lu27	04:01	04:01//04:01	2
	12lu43	04:01	04:01//04:01	2
Ossabaw	466	08:04/11:04	08:04//11:04	2
	556	08:04/08:16	08:04//08:16	2
	332	08:16	08:16//08:16	2
	337	08:04/11:04/21:02/23:03	08:04//11:04//21:02//23:03	4
	344	08:04/11:04	08:04//11:04	2
American Guinea Hog	F=Frisky	04:01/07:07	04:01//07:07	2
	D=Delifay	16:01/07:07	16:01//07:07	2
Meishan	10me4 (m135)	04:01	04:01//04:01	2
	10me7 (m2782)	13:02/17:01/08:13/10:02	10:02-17:01//08:13-13:02	4
	10me3 (m89)	04:01/10:02/17:01	04:01//10:02-17:01	3
	10me2 (m67)	02:01/02:02/04:01/15:03	02:01//02:02//04:01//15:03	4
ATCC cell	CCL-166	02:01/07:01/11:04	02:01-07:01//11:04	3
	CRL-1746	07:02/07:05	07:02//07:05	2
	CL-184	14:01/14:02	14:01//14:02	2
	CRL-2528	12:01/13:01/08:07	12:01-13:01//08:07	3
	CRL-2842	02:02/08:08/18:01	02:02-18:01//08:08	3
	CRL-6489	04:01	04:01//04:01	2
	CL-101	11:01	11:01//11:01	2
	CCL-33	04:01	04:01//04:01	2
Local PAM cell	P31	07:02/11:02	07:02//11:02	2
	P39	08:17/11:02	11:02//08:17	2
	P61	04:02	04:02//04:02	2
	P303	08:01/08:10	08:01//0810	2
Land x KNP	J16-2	08:01/11:02	08:01//11:02	2
	J16-3	08:01/11:02	08:01//11:02	2
	J16-4	11:03/21:01//15:01/09:01	11:03-21:01//15:01-09:01	4
	L83-2	11:02/14:01	11:02//14:01	2
	L83-3	14:01/15:02	14:01//15:02	2
	L83-5	14:01/15:02	14:01//15:02	2

Table S2. Family segregation analysis of *SLA-1* typing using 11 families with pedigree information.

Family	Father	SLA1	Mother	SLA1	Offspring	SLA1
1	J-3019	08:01/11:02	J-2013	11:02/12:01	J-4003	08:01/11:02
					J-4004	08:01/11:02
					J-4006	08:01/11:02
2	J-3017	11:02/15:02	J-3013	11:02	J-4008	11:02/15:02
3	J-3017	11:02/15:02	J-3020	11:02/15:02	J-4012	15:02
					J-4014	11:02/15:02
					J-4015	15:02
4	J16-33	11:02/(11:03/21:01)	L5-68	08:01/(15:01/23:03)	J16-2	08:01/11:02
					J16-3	08:01/11:02
					J16-4	11:03/21:01//15:01/23:03
5	K27	11:02/15:02	L5-83	08:11/14:01	L83-2	11:02/14:01
					L83-3	14:01/15:02
					L83-5	14:01/15:02
6	J-105	08:01/11:02	J-2029	08:01	J-3001	08:01
7	J-105	08:01/11:02	J-2001	08:01/12:01	J-3007	08:01
					J-3008	11:02/12:01
8	K-27	11:02/15:02	J-2038	11:02/15:02	J-3012	11:02/15:02
					J-3013	15:02
					J-3014	11:02/15:02
					J-3015	15:02
					J-3016	11:02
					J-3017	11:02/15:02
9	K-27	11:02/15:02	J-2008	08:01/11:02	J-3018	08:01/11:02
					J-3019	08:01/11:02
					J-3020	11:02/15:02
					J-3021	11:02
10	J-105	08:01/11:02	J-2001	08:01/12:01	J-3022	08:01/11:02
					J-3023	11:02/12:01
					J-3024	11:02/12:01

					J-3025	08:01
					J-3026	08:01/11:02
					J-3027	11:02/12:01
11	J-105	08:01/11:02	J-2013	11:02/12:01	J-3028	11:02/12:01
					J-3029	08:01/11:02
					J-3030	08:01/11:02
					J-3031	08:01/11:02
					J-3032	11:02
					J-3034	11:02

Table S3. Comparison of *SLA-1* typing results from cell lines between the genomic- and cDNA-based typing.

	Cell lines	Origin	cDNA typing	gDNA typing	*RT-PCR and cloning
ATCC	CCL-166	kidney	02:01/07:01/11:04	02:01/07:01/11:04	02:01/07:01/11:04
	CRL-1746	testis	07:02/07:05	07:02/07:05	07:02/07:05
	CL-184	kidney	14:01/14:02	14:02/14:01	14:02/14:01
	CRL-2528	nasal turbinate/mucosa	12:01/13:01/08:07	12:01/13:01/08:07	12:01/13:01/08:07
	CRL-2842	kidney cortex	02:02/08:08/18:01	02:02/08:08/18:01	02:02/08:08/18:01
	CRL-6489	kidney	04:01	04:01	04:01
	LLC-PK1	kidney	11:01	11:01	11:01
	CCL-33	kidney	04:01	04:01	04:01
Local PAM cells	P31	Alveolar macrophages	07:02/11:02	07:02/11:02	
	P39		08:17/11:02	08:17/11:02	
	P61		04:02	04:02	
	P303		08:01/08:10	08:01/08:10	

* Previous typing results from Ho et. al., 2009.

Table S4. Novel *SLA-1* alleles identified in the study.

Official name	Accession	Breeds	Number of individuals
SLA-1*07:03	[b]KU754555.1	Yorkshire, Sam's, Chonbuk	6
SLA-1*07:07	MF871653	AGH	2
SLA-1*07:08	MF871650	Yorkshire	4
SLA-1*08:15	[b]KJ555020.1	Berkshire	6
SLA-1*08:16	MF871654	Ossabaw	2
SLA-1*08:17	[a] MF871647	Duroc, Landrace, local PAM cells, Chonbuk	24
SLA-1*15:03	[b]MF498783.1	Berkshire, Meishan, Sam's	7
SLA-1*16:01	MF871646	Duroc, AGH, Chonbuk	25
SLA-1*18:02	[b]AB845314	SNU	8
SLA-1*19:02	MF871652	Lanyu	1
SLA-1*20:02	MF871649	Landrace, Sam's	2
SLA-1*21:01	[b]KU754556.1	Yorkshire, KNP, Land x KNP, Chonbuk	19
SLA-1*21:02	MF871648	Berkshire, Ossabaw	11
SLA-1*21:03	[b]AK394788	Yorkshire, Landrace	3
SLA-1*23:01	[b]KF026021	Landrace	4
SLA-1*23:02	MF871651	Berkshire	10
SLA-1*23:03	MF871655	Ossabaw	1
SLA-1*23:04	[b]KJ555027.1	Berkshire, Yorkshire	7

[a] Transcript confirmed by amplification from cDNA in this study.

[b] alleles previously identified, but not submitted to IPD.

Table S5. Pairwise comparison analysis of genetic identity among different breeds of pigs

Breed	KNP	SNU	NIH	Duroc	Berkshire	Yorkshire	Landrace	ATCC	Land x KNP	Lanyu	Ossabaw	Meishan	Local PAM cell	AGH
**KNP (114)**	**1.000**													
**SNU (52)**	**0.000**	**1.000**												
**NIH (29)**	**0.000**	**0.964**	**1.000**											
**Duroc (21)**	**0.049**	**0.000**	**0.213**	**1.000**										
**Berkshire (19)**	**0.000**	**0.000**	**0.011**	**0.049**	**1.000**									
**Yorkshire (19)**	**0.070**	**0.000**	**0.031**	**0.138**	**0.038**	**1.000**								
**Landrace (19)**	**0.159**	**0.000**	**0.034**	**0.172**	**0.077**	**0.172**	**1.000**							
**ATCC (8)**	0.022	0.118	0.265	0.663	0.132	0.359	0.291	1.000						
**Land x KNP (6)**	0.755	0.000	0.000	0.034	0.027	0.091	0.615	0.093	1.000					
**Lanyu (5)**	0.000	0.000	0.218	0.967	0.049	0.141	0.156	0.672	0.000	1.000				
**Ossabaw (5)**	0.000	0.000	0.000	0.000	0.196	0.000	0.078	0.085	0.000	0.000	1.000			
**Meishan (4)**	0.000	0.000	0.158	0.699	0.026	0.204	0.113	0.607	0.000	0.714	0.000	1.000		
**Local PAM cell (4)**	0.608	0.000	0.000	0.109	0.071	0.164	0.113	0.049	0.422	0.000	0.000	0.000	1.000	
**AGH (2)**	0.000	0.000	0.090	0.410	0.020	0.058	0.064	0.276	0.000	0.406	0.000	0.293	0.000	1.000

Note: Genetic identities were calculated following the Nei's method. $I^{Nei} = Jxy/(JxJy)^{0.5}$. Where Jx is the sum of the squared allele frequencies for population x; Jy is the sum of the squared allele frequencies for population y; Jxy is the sum of the products of allele frequency. The calculation for sample sets with number of pigs less than 10 could involve bias from sample size.

Table S6. List of *SLA-1* alleles shared by multiple breeds of pigs.

Number of breeds with allele sharing	Alleles	Breeds
5	04:01	NIH, Duroc, Berkshire, Yorkshire, Landrace
3	07:02	Duroc, Berkshire, Landrace
	12:01	KNP, Yorkshire, Landrace
2	01:01<br>08:01<br>08:11<br>13:01<br>21:03	Yorkshire, Landrace
	08:01<br>15:01<br>15:02	KNP, Landrace
	11:03<br>21:01	KNP, Yorkshire
	11:01(01|02)<br>23:04	Berkshire, Yorkshire
	11:04<br>14:01	Berkshire, Landrace
	08:17	Duroc, Landrace
	11:02	KNP, Duroc

Table S7. The list of pig breed-specific alleles for *SLA-1*.

Breeds	Alleles
Yorkshire	02:02, 07:04, 08:08, 18:01, 07:03, 07:08
Landrace	08:03, 08:05, 09:01, 20:02, 23:01
Berkshire	04:02, 08:15, 21:02, 15:03, 23:02
NIH	02:01, 07:01
Duroc	16:01
